# Analysis of Interactions Between Pyomelanin and the Extracellular Matrix in an Ex Vivo Turkey Tendon Model

**DOI:** 10.1002/open.202500194

**Published:** 2025-04-13

**Authors:** Rebecca F. Shepherd, Hanaa A. Galeb, Jade Bentham, Reza Moshrefi, Katelyn Ryan, Nur Adeelah Che Ahmad Tantowi, Sara J. Baldock, Nathan R. Halcovitch, T. Jane Stockmann, John G. Hardy, Jemma G. Kerns, Adam M. Taylor

**Affiliations:** ^1^ Lancaster Medical School Lancaster University Lancaster LA1 4AT UK; ^2^ Department of Chemistry, Science & Arts College, Rabigh Campus King Abdulaziz University Rabigh 25732 Saudi Arabia; ^3^ Department of Chemistry Lancaster University Lancaster LA1 4YB UK; ^4^ Department of Chemistry Memorial University of Newfoundland 45 Arctic Ave St. John's NL A1C 5S7 Canada; ^5^ Department of Economics and Agribusiness School of Economics, Finance and Banking Universiti Utara Malaysia 06010 Sintok Kedah Darul Aman Malaysia

**Keywords:** alkaptonuria, extracellular matrix, pyomelanin, scanning electrochemical microscopy, spectroscopy

## Abstract

Melanins are conjugated biopolymers with varying compositions and functions, found in various tissues throughout the body. Here, the conjugated polymers derived from homogentisic acid (HGA), polyHGA (a simplified model of pyomelanin), formed in an ex vivo tendon model are examined with a view to understanding interactions between melanins and the extracellular matrix (ECM) using a selection of different analytical techniques, including spectroscopy (energy dispersive X‐ray, infrared, and Raman), X‐ray diffraction, and microscopy (electron, optical, and scanning electrochemical). The combination of techniques was used to facilitate an understanding of subtle differences in the composition and distribution of ECM components, hydroxyapatite, and melanin in the tendons for the first time. PolyHGA deposition in connective tissues in patients with alkaptonuria is a significant burden and causes multiple tendon ruptures due to the significant alterations in collagen properties. A similar pathology is seen in the wider population from calcific tendinitis because of hydroxyapatite crystal deposition in tendons of the shoulder and lower limbs, in particular.

## Graphical abstract

Polymerization of homogentisic acid in the presence of extracellular matrix (ECM) components (an ex vivo turkey tendon model) yields simplified versions of biologically generated melanins in the ECM, facilitating studies of melanin–ECM interactions. A variety of techniques (X‐ray diffraction, microscopy, and spectroscopy) were employed to understand the potential influence of the ECM in the deposition of melanins in biological tissues.

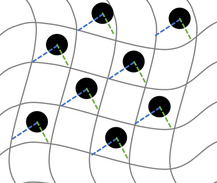

## 1 Introduction

Melanins are a ubiquitous class of biopolymers that form colored pigments. Their variable chemical structures and physical properties and the conjugation of their backbones result in semicrystallinity and interesting optoelectronic properties which are of interest to researchers in academia and industry. A number of literature reviews have been written about this class of biopolymers, exploring the polymerization of the monomers (i.e., phenolic, indolic, or dihydroxynaphthalene precursors), structure‐function relationships, and their potential technical and medical applications.^[^ ^1^, ^2^, ^3^, ^4^, ^5^, ^6^, ^7^ ^]^

The two types of melanins commonly responsible for the coloration of the skin and hair are eumelanin (a brown to black‐colored pigment, the precursor for which is levodopa [DOPA]) and pheomelanin (a yellow to red pigment, the precursors for which are DOPA and the amino acid cysteine),^[^ ^8^, ^9^ ^]^ produced by the Raper–Mason scheme.^[^ ^10^, ^11^ ^]^ Neuromelanin is another type of melanin (a black‐colored pigment, the precursor for which is DOPA) produced in the substantia nigra and locus coeruleus. Pyomelanin is another type of melanin (a black‐colored pigment, the precursor for which is homogentisic acid (HGA)) produced in patients with alkaptonuria (AKU).^[^ ^12^, ^13^, ^14^, ^15^, ^16^, ^17^, ^18^ ^]^

Many potential purposes or functions of melanins in the extracellular environment have been suggested, and their extracellular presence could have beneficial or detrimental consequences.^[^ ^19^, ^20^ ^]^ Melanins can adhere to a wide variety of biomolecules found in the biological milieu, with important biologically relevant roles;^[^ ^21^, ^22^, ^23^ ^]^ due to the adhesive nature of the functional groups in the melanins (e.g., catechols).

Melanin in healthy tissues is typically found in specialized cells or areas of the body, such as melanocytes of the skin or the substantia nigra in the brain. Melanin is normally not associated with ECM proteins, although a melanin like polymer, polyHGA, has been show to associate with ECM proteins such as collagen and GAGs of the ECM in AKU.^[^ ^24^, ^25^ ^]^ While melanin is not typically found in the ECM of healthy tissues, its precursor—tyrosine—and the intermediate dopaquinone, as well as other polyphenol and quinones, have shown the ability to bind collagen and stiffen it.^[^ ^26^ ^]^ While stiffer collagen is beneficial in some instances, it is a challenge to get the level of stiffness correct, given that overly stiff collagen alters mechanical loading through the tissue(s), often pathologically through stress shielding in tendons and bone,^[^ ^27^, ^28^, ^29^, ^30^, ^31^, ^32^ ^]^ as well as through fibrosis in lung tissues.^[^ ^33^ ^]^

Phenolic monomers are common building blocks of melanins (observed in allomelanin, eumelanin, neuromelanin, pheomelanin, and pyomelanin)^[^ ^34^ ^]^ and are known to play important roles in intermolecular/material interactions.^[^ ^35^, ^36^, ^37^ ^]^ In continuation of our studies on pyomelanin,^[^ ^38^, ^39^ ^]^ here we examine the properties of conjugated polymers derived from phenolic monomer HGA polymerized in the presence of ECM components from Turkey tendon ex vivo. A selection of different analytical techniques was employed for these studies, including histology, Raman spectroscopy, X‐ray diffraction (XRD), Fourier‐transform infrared (FTIR) spectroscopy, scanning electron microscopy (SEM), energy dispersive X‐ray (EDX) spectroscopy, and scanning electrochemical microscopy (SECM). The properties of the materials offer insights into the role of functional groups pendant on the polymer backbones on melanin–ECM interactions using simple model systems (polyHGA‐tendon in this case).

## 2 Results and Discussion

**Scheme 1:**
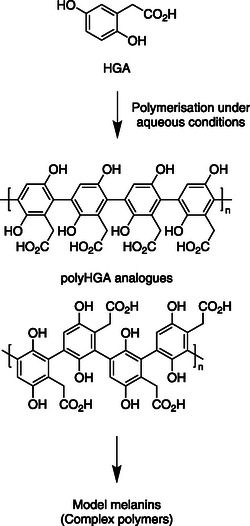
A schematic of the polymerization of HGA to form polyHGA, a simplified model of pyomelanin studied herein. It is important to note that natural pyomelanin samples may contain other monomers depending on the conditions under which they are formed in vivo.

The predominant type of collagen observed in tendons is type I collagen, with small amounts of other types of collagen present with distributions that vary throughout the tendon impacting their physicochemical properties and concomitantly mineralization and mechanics.^[^ ^40^, ^41^, ^42^, ^43^ ^]^ In silico and experimental studies have demonstrated collagen–hydroxyapatite interactions to be governed by the polar functional groups displayed on collagen (e.g., ─OH, ─NH_3_^+^, CO_2_^−^) which bind strongly to the surface of hydroxyapatite,^[^ ^44^, ^45^, ^46^, ^47^ ^]^ and it is therefore possible that subtle differences in the isoelectric points of the various types of collagen and their distributions in the ECM of tissues will concomitantly affect the distribution of mineralization and melanins therein. Consequently, with a view to understand pyomelanin–ECM interactions (e.g., potential role of the ECM in controlling the distribution of melanin in tissues, particularly as pyomelanin is anionic at physiological pH), HGA was polymerized via autooxidation (**Scheme** **1**) in the presence of the ECM components (ex vivo Turkey tendon model). The polymerization of HGA in the tendon resulted in the coloration of the tendon due to the formation of ochronotic pigment due to the presence of nanoscale particles of 10–100 s of nm composed of conjugated polymers (the formation of which is proposed to proceed via a nucleation and growth mechanism)^[^ ^48^ ^]^ that may further aggregate to larger structures.^[^ ^49^ ^]^ The presence of these small 10–100 s of nm conjugated polymers represents the detection of the smallest amounts of polymerized pigments in experimental models to date. These pieces are significant because they are present after only a few days, rather than the years it is believed for the pigment to begin to be present in AKU patient tissues. Further work is needed to determine the earliest point at which these minute particulates may be present in a patient's tissues. This is fundamentally important in understanding the pathological processes in AKU patient tissues to enable the targeting of any therapy at the earliest point prior to alteration of the ECM by any nm‐scale diameter particles. This coloration was consistent with the ochronotic description of AKU tissues in the literature at the macroscopic, microscopic, and ultrastructural levels.^[^ ^16^, ^31^ ^]^ TEM examination has shown dark crystalline‐like depositions within the collagen fibers, between them and protruding from them out into the ECM, creating visible and atomically detectable disorder in the ECM in AKU patient tissues.^[^ ^25^, ^50^ ^]^

### 2.1 Gross and Histological Studies

**Figure 1:**
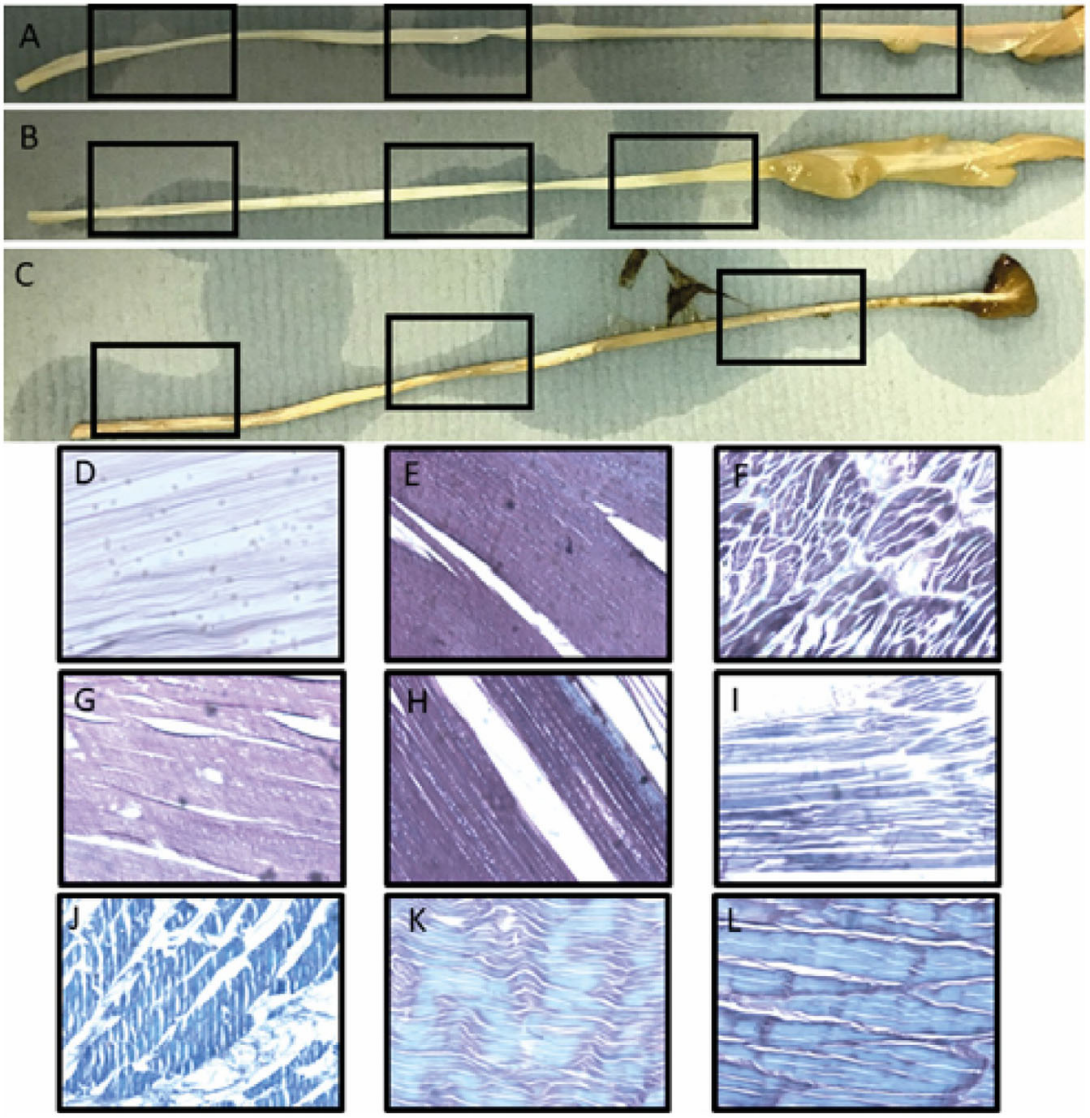
Macroscopic and histological images (×10 magnification) of turkey tendons incubated in HGA; A) control PBS, insets are histology locations at D) distal, E) middle, and F) proximal segments. B) Low conc HGA; 0.33 μM, insets are histology locations G) distal, H) middle, and I) proximal. C) High conc HGA; 0.33 mM, insets are J) distal, K) middle, and L) proximal. High‐concentration HGA‐soaked tendons show positive Schmorl's (blue) staining indicative of polyHGA, with low concentrations showing smaller amounts at the periphery and absence in control culture sections.

The tendons studied herein displayed clear differences in their culture PBS medium, with the higher concentrations displaying darker coloration than the low HGA media after 7 days (**Figure** **1**). Macroscopic observation of the highest HGA concentration reveals distinct dark coloration, consistent with ochronosis of AKU patient tissues; this was most marked at the proximal end of the tendon. The lowest concentration of HGA‐soaked tendon displayed similar ochronotic discoloration but to a lesser extent than the higher concentration. There was no ochronotic coloration of tendon or discoloration of media in the control tendon.

Histological analysis of tendons and their regions revealed distinct and strong Schmorl's stain (blue coloration), consistent with the presence of melanin or polyHGA in samples. This was seen to a lesser extent in low HGA concentration and absent from the controls, with counterstain being clearly observed and absence of blue coloration, indicative of polyHGA. The densest staining indicating the presence of HGA was at the proximal end, followed by distal and then middle. This is consistent with the macroscopic appearance of the tendons at day 7.

The presence of HGA pigment in the proximal end, which is the most mineralized part, is interesting given that in mature mineralized bone, it is thought that the mineral protects the collagen fibers from binding by HGA/polyHGA, but in this model, it appears that relatively immature mineralized collagen shows binding by polyHGA. The middle third of the tendons at both concentrations show the most notable amount of pigmentation from Schmorl's staining, consistent with Raman and XRD data demonstrating the highest amount of collagen here; this could represent the location of most “nucleation sites” on each of the collagen fibers, consistent with data already observed by TEM.^[^ ^25^ ^]^

### 2.2 Raman Spectroscopy and XRD

**Figure 2:**
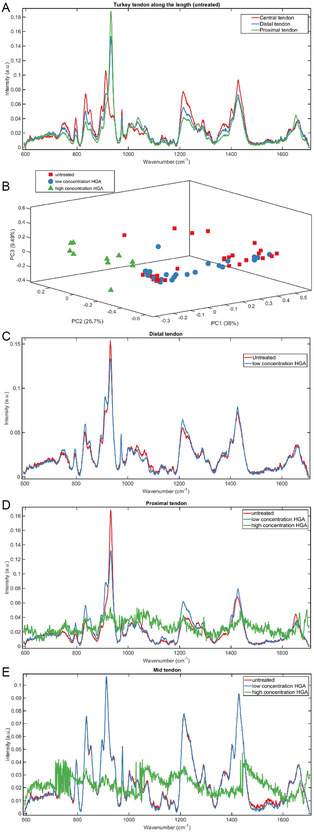
Raman spectra of the tendons: A) average spectra from each third of the tendons; B) PCA scores plot of the untreated (red rectangles), low‐concentration HGA (blue circles) and high‐concentration HGA (green triangles); C) average spectra of the distal tendon by treatment; D) average spectra of the proximal tendon by treatment; and E) average spectra of the middle tendon by treatment.

Raman spectra of samples are shown in **Figure** **2**A–E. The average Raman spectrum from the untreated (control) tendons indicates that, although the turkeys were 20 weeks old, the mineralization status of the distal ends of the tendons is incomplete. Specifically, from 12 to 18 weeks of age, the tendons mineralize at the proximal and then distal ends, leaving the central third nonmineralized. Across all the spectra (Figure 2A,C–E) from the control and low‐concentration treated tendons of all regions, the spectra are characteristic collagen spectra, with specific peaks at 1650 cm^−1^ (amide I), 1450 cm^−1^ (CH_2_), 1240–1260 cm^−1^ (amide III), 920 cm^−1^ (proline), and 870 cm^−1^ (hydroxyproline). Further, the spectra in Figure 2A have a clear phosphate peak (960 cm^−1^) in the proximal spectra, no phosphate peak in the central third, and a developing peak in the distal third; likewise, the XRD patterns (Figure S1, Supporting Information) for the untreated control tendons show peaks between 2*θ* 31° and 33° characteristic of hydroxyapatite for the proximal and distal samples, but not the midpoint. Interestingly, nonmineralized collagen (evident from the distinct proline peak at 920 cm^−1^ and lack of phosphate peak in the Raman spectra) is more distinct in the distal compared to the proximal spectra; furthermore, the relative amount of collagen present is higher in the central third compared to either end. PCA analysis (Figure 2B) of the untreated compared to the two HGA‐treated groups reveals that the high concentration is more distinct and separate from the lower concentration and untreated groups. As is demonstrated from the spectra in Figure 2D,E, the presence of high‐concentration HGA led to the collection of spectra with a low signal‐to‐noise ratio, with none being able to be collected from the treated distal tendon. This is consistent with spectra obtained when comparing ochronotic vs nonochronotic cartilages from AKU patients showing differences in spectra from each other and compared to “normal” cartilage.^[^ ^51^ ^]^ At the point of spectral collection, the high concentration of treated tendons resulted in a saturated detector, that is, a large amount of light flooding the detector, meaning that no measurements could be acquired. A comparison of what is happening at each of the three areas of the tendon shows that the effect of the lower concentration HGA on the distal (Figure 2C) third of the tendons decreases the mineral content relative to controls. This is also demonstrated in the proximal third (Figure 2D) of the tendons, to a higher extent. In the central third (Figure 2E), which did not contain mineral, there was less of an effect, with the spectra visually comparable. Due to the number of samples, further multivariate analysis was not possible. However, a comparison of 1/FWHH revealed that the untreated proximal and distal spectra were more mature, that is, had a higher degree of crystallinity, compared to the tendons treated with a low concentration of HGA (data available on request). This observation is supported by XRD data, noting that XRD patterns of the samples (Figure S1, Supporting Information) suggested that with the exception of hydroxyapatite, the samples were largely amorphous at the microscale (i.e., molecular lattice, unit cell dimension symmetry), characteristic of collagen and natural/synthetic melanins.^[^ ^52^, ^53^, ^54^, ^55^, ^56^, ^57^ ^]^ Overall, the treatment of the tendons with a high concentration of HGA was disruptive to the tendons to the point where measurements could not be equally acquired from all tendons. A comparison of untreated to low‐concentration HGA treatment shows that in areas of the tendons that are mineralized, the mineral components are reduced and less mature, potentially due to the HGA either blocking the mineral or disrupting it (supported by XRD data). In the central region, there was less of an effect; however, this warrants further investigation with more samples, as it is known that naturally nonmineralized collagen is affected in AKU, the condition where people have an excess of HGA.

### 2.3 FTIR Spectroscopy

**Figure 3:**
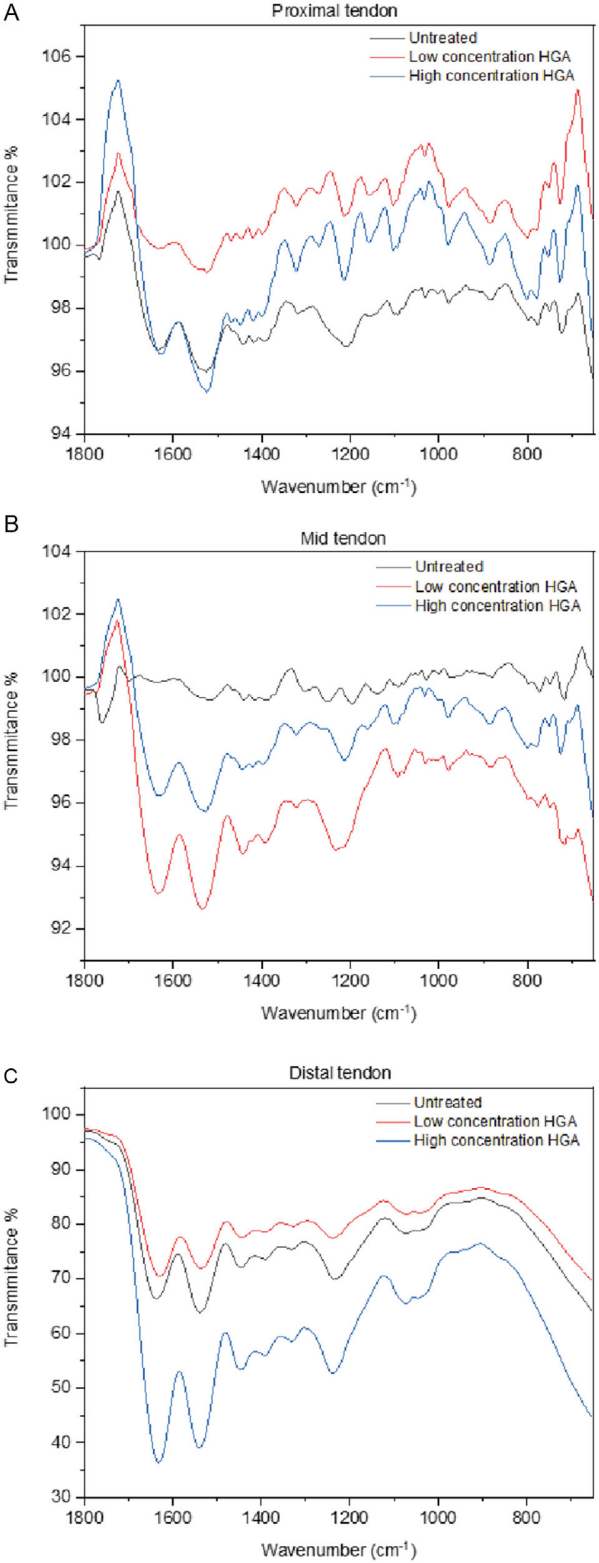
FTIR spectra of tendons. A) Proximal section of tendon. B) Midpoint of tendon. C) Distal section of tendon.

FTIR spectroscopy was used to analyze the samples studied herein which were complex due to the variety of chemical environments (**Figure** **3**, Figure S2, Supporting Information) and insights compared to conclusions drawn from the Raman data.^[^ ^58^, ^59^, ^60^, ^61^, ^62^, ^63^, ^64^, ^65^ ^]^ The spectra for all samples showed peaks characteristic of the ECM components in tendon, particularly the amide I (1620–1680 cm^−1^), amide II (1510–1560 cm^−1^), amide III (1190–1320 cm^−1^), amide A (3305 cm^−1^), and amide B (2936 cm^−1^) bands from the proteinaceous content and peaks characteristic of arginine (a strong absorption between 1688 and 1695 cm^−1^ from ν_as_(CN_3_H_5_^+^) and a weaker absorption at 1576–1577 cm^−1^ from v_s_(CN_3_H_5_^+^)); however, these may overlap with amide I and II peaks: peaks characteristic of proline (at 1456 cm^−1^ from ðCH_2_ and at ≈1400–1454 cm^−1^ from νCN) and peaks characteristic of aspartic acid (at 1729 cm^−1^ from ν(C=O) of the carboxylic acid and at 1570 cm^−1^ from ν_as_(COO^−^)). While it is plausible the peak between 850 and 890 cm^−1^ corresponds to the ν_2_ carbonate region (particularly at 884 cm^−1^), and the peak between 900 and 1180 cm^−1^ corresponds to the ν_1_ and ν_3_ phosphate region of apatite (particularly at 978 and 1030 cm^−1^), it is possible that the peaks are from other functional groups in the samples (e.g., aromatic C─H bonds). It is noteworthy that while it is easy to visually assess the presence of polyHGA in samples due to their coloration (i.e., tendon incubated with low and high concentrations of HGA), the spectra are convoluted due to overlapping peaks in the region corresponding to phenolic OH and aromatic C=C bonds, at ≈1200–1210, ≈1560–1570, and 1500–1510 cm^−1^, respectively. Importantly, the peaks from the polyHGA are most clearly evident in the middle third of the tendons (≈1560–1570 and 1500–1510 cm^−1^, in samples exposed to both low/high concentrations of HGA) which also show the most notable amount of pigmentation from Schmorl's staining (characteristic of melanins). The overlapping peaks can make it difficult to draw conclusions about pyomelanin–ECM or pyomelanin–mineral interactions; however, akin to the Raman studies, the treatment of the tendons with a high concentration of HGA was disruptive to the tendons, with observable differences in peak shape and intensity for the amide I, amide II, amide III, amide A, and amide B peaks. Likewise, Raman spectra show that nonmineralized collagen is more distinct in the distal compared to the proximal region, which is supported by the FTIR data showing relatively clear peaks at 978 and 1030 cm^−1^ (characteristic of phosphates in apatite) in the spectra from the proximal region.

### 2.4 SEM‐EDX

SEM‐EDX images of the samples (Figure S3–S11, Supporting Information) shows a degree of organization of the collagen fibrils characteristic of tendon tissue,^[^ ^66^, ^67^ ^]^ and exposure to high concentrations of HGA and concomitant polyHGA deposition disrupts this organization, as previously seen in collagen fibers from other AKU connective tissues, where collagen structure is compromised and alterations in the structural arrangement of tissues is seen.^[^ ^25^, ^50^, ^51^ ^]^ EDX data suggests that all samples are mainly composed of C, N, O, P, and S characteristic of proteins and other biomolecules, with additional traces of Cl, K, Na, and P from the buffer (Figure S3–S11, Supporting Information); however, quantitative comparison was not possible.

### 2.5 SECM

**Figure 4:**
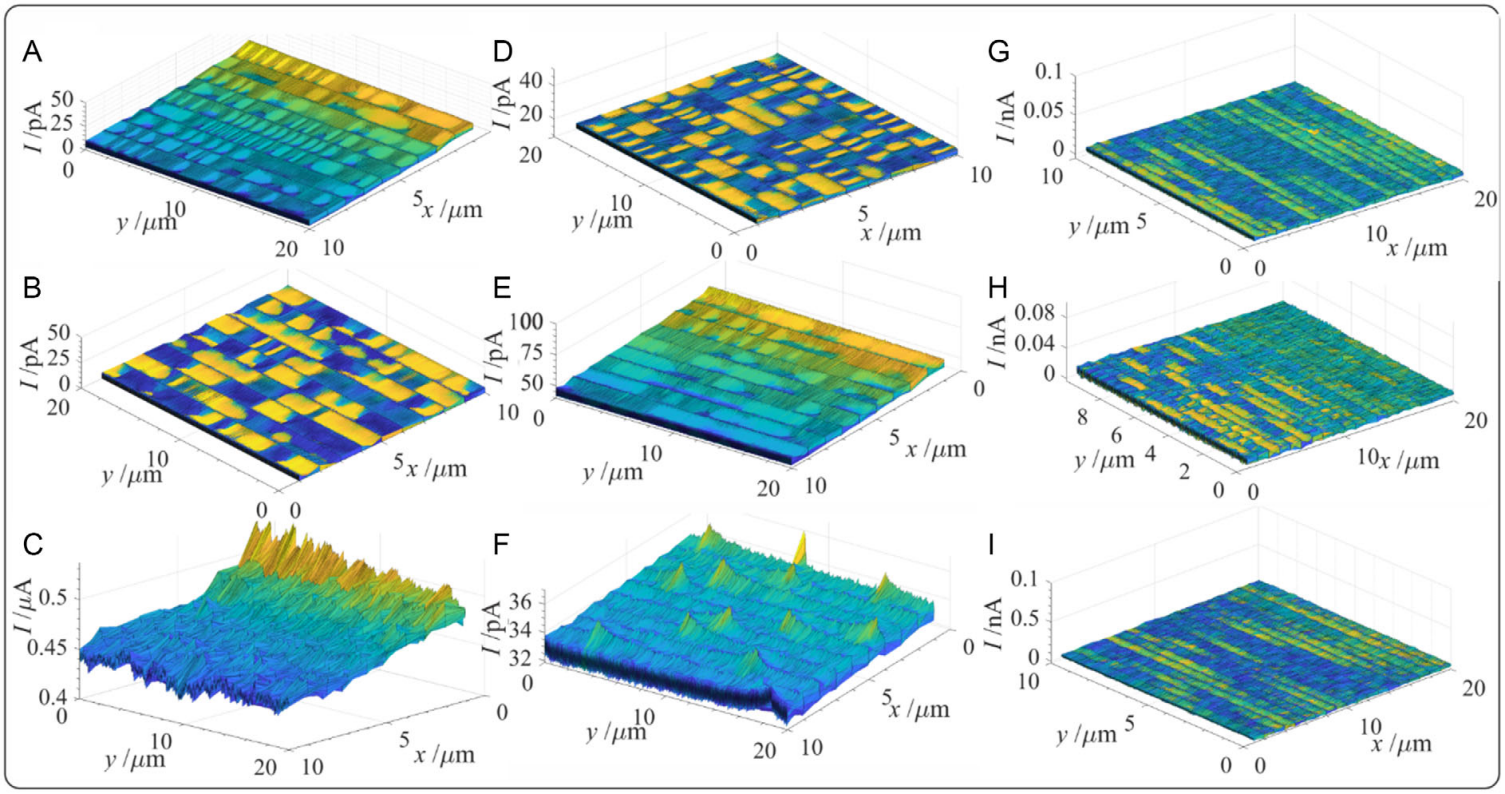
SECM images obtained using a 7 μm diameter carbon fiber disc UME in 2‐electrode, constant height mode (1 μm tip‐to‐substrate distance) using 0.9 mM ferrocene methanol (FcCH_2_OH) as a redox mediator and 0.1 m NaCl as a supporting electrolyte. An Ag wire was employed as the counter electrode and the tip was moved at a rate of 1 μm s^−1^. An FcCH_2_OH oxidizing potential was determined using CV (data not shown) and maintained throughout. The A–C) left, D–F) middle, and G–I) right‐hand columns correspond to proximal, midpoint, and distal sample locations, while the (A,D,G) top, (B,E,H) middle, and (C,F,I) bottom rows are control, low, and high exposure samples, respectively.

Scanning probe microscopy uses various probes to analyze the surface of samples enabling examination of a multitude of properties, and SECM enables studies of electrochemical processes on a variety of surfaces, including for biological samples.^[^ ^68^, ^69^, ^70^, ^71^, ^72^ ^]^ SECM shows differences in conductivity between the samples (**Figure** **4**). Melanin nanoparticles have demonstrated unique electrical conductivity properties and ζ‐potentials in the range of −40 mV.^[^ ^73^, ^74^, ^75^ ^]^ Moreover, they have also been shown to possess antioxidant properties. Tendon samples were fixed to a glass slide using a two‐part epoxy resin, which was allowed to dry overnight. These samples are 3D and contoured with a variable topography; however, reasonably flat regions were identified using an optical microscope (90× objective) and probed via SECM using a 7 μm diameter inlaid disc ultramicroelectrode (UME) with 0.9 mM ferrocene methanol (FcCH_2_OH) as a redox mediator over a relatively small area, 10 × 20 μm (details regarding UME characterization are in the experimental section and Figure S12, Supporting Information). Probe approach curves (PACs), that is, advancing the UME tip towards the substrate in the *z*‐direction, were performed until an insulating, negative feedback response was observed. Thus, the bulk tissue showed insulating behavior or was inert towards the FcCH_2_OH redox mediator. Subsequently, the UME tip was retracted to a *z*‐position of roughly 1 μm away from the substrate, and the UME was then rastered back and forth in the *x*‐*y* plane to generate the electroactivity maps shown in Figure 4. All samples demonstrated a low background tip current in the picoampere (pA) regime with little observable change in topography. Control samples or those prepared with a low biomimetic concentration of HGA elicited no distinguishable changes in electroactivity. However, electrochemical images taken at proximal and distal samples showed current spikes indicative of positive feedback (Figure 4C,F). These current spikes, particularly in Figure 4F, are discernibly more conductive which we ascribe to the presence of polyHGA particles embedded in the tendons. Figure 4C shows the electroactivity response at high HGA in the proximal sample and demonstrates an odd, massive current increase (μA range) across the entire image. While poorly reproducible, this may point to a widespread distribution of smaller melanin nanoparticles/nanoclusters/aggregates of polyHGA or loss of redox active melanin molecules to the solution.

The pigmentation of the melanins in the ECM is suggestive of various lengths of melanin polymers and various oxidation states of the monomeric units constituting the backbone of the melanins. Contributions from intramolecular and intermolecular melanin–melanin interactions and melanin–ECM interactions will contribute towards the hierarchical assembly of the melanins (e.g., π‐stacking interactions which play a role in melanin aggregation) and the assembly of the ECM, concomitantly on the electronic properties of the melanin–ECM composites and the electrophysiology of the tissues.

## 3 Conclusions

Herein, polymerization of an HGA in the presence of ECM components (an ex vivo turkey tendon model) yielded conjugated polymer‐based materials polyHGA in ECM. A variety of analytical techniques (histology, Raman, XRD, FTIR, SEM‐EDX, and SECM) were employed to examine the composites produced. The experiments revealed interesting trends in the distribution of ECM/hydroxyapatite/poly/HGA at different points in the tendons, with SECM offering insights into the conductivity of the tissues which may play a role in their electrophysiology.^[^ ^76^ ^]^ HGA concentration is elevated in patients with AKU and causes widespread systemic pathologies through the polyHGA deposits. The conductivity of polyHGA is a novel finding in this study and raises questions about the involvement it may have in neurological role(s) in AKU. There is currently no evidence of significant differences in nerve conduction speeds in patients with AKU compared to normal/control subjects; this could partly be due to the limited number of AKU patients able to participate due to the rarity of the condition but likely warrants further investigation,^[^ ^76^ ^]^ AKU patients have been detailed with Parkinson's disease in the medical literature; it is not clear whether this is above the presence in the wider non‐AKU population.^[^ ^77^, ^78^ ^]^ There is also a growing body of literature around the presence of ochronotic pigment in neurological tissues and those that surround them, raising the question of whether there is any role of HGA or polyHGA in the nervous system and presentation of multiple neurological symptoms that AKU patients present with clinically.^[^ ^77^, ^79^, ^80^, ^81^ ^]^ Furthermore, collagen and collagen‐hydroxyapatite composite‐based biomaterials are interesting for biomedical applications,^[^ ^63^, ^82^, ^83^, ^84^ ^]^ as indeed are electroactive biomaterials,^[^ ^85^, ^86^ ^]^ and it is conceivable that analogous biomineralized electroactive materials may be of interest as instructive biomaterials for tissue engineering and regenerative medicine.^[^ ^87^, ^88^ ^]^

## 4 Experimental Section

4.1

4.1.1

##### Materials

Unless otherwise noted, all chemicals and consumables were supplied by Sigma Aldrich (Merck), Gillingham, UK.

##### Preparation of Samples

Two turkeys (*Meleagris gallopavo* domesticus), aged 20 weeks, were purchased from a farm and killed in accordance with UK food and farming standards. Prior to dissection, the turkeys were stored at −20 °C. *M. gallopavo* flexor hallucis longus and *M. gallopavo* flexor digitorum were targeted for removal from each leg, as they are the longest tendons present in the leg of the turkey. The tendons were placed in solutions of 0.33 and 0.33 mM and a control solution of PBS and incubated at 37 °C for 7 days. Note regarding ethical approval: all samples were collected in accordance with UK food and farming standards.

##### Histology

Tendons were fixed in formalin for 3 days and then dissected into 1 cm lengths from proximal to distal and processed for routine histology by paraffin embedding. Four micrometer sections were cut and stained with either H&E or Schmorl's reagent. Sections were dehydrated through graded alcohols and mounted in DPX, as previously described.^[^ ^50^ ^]^ Microscopy was conducted using a Nikon Eclipse 50i with an attached Nikon Digital Sight camera.

##### Raman Spectroscopy

Raman spectra were acquired from the tendons using an InVia Raman microspectrometer (Renishaw Ltd), equipped with a 785 nm laser. Spectra were acquired at 100% laser power (2 mW at sample) at 30 s and 2 accumulations. Prior to spectral acquisition, tendons were thawed at ambient temperature. Data was collected from the tendons in the same orientation, acquiring spectra from the distal to proximal end. An origin was set 1 cm superior to the bifurcation point (distal end) of the tendon. Spectra were collected along the length of each tendon at 10 mm intervals, with the spectra labeled as belonging to the proximal, middle, or distal thirds for subsequent analysis. All spectra were baseline corrected using polynomial order 7 and vector normalized. Principal component analysis (PCA) was performed using an in‐house written MATLAB script (MATLAB, The Math‐Works, Inc.),^[^ ^89^, ^90^ ^]^ which is an unsupervised technique that forms linear components and considers the data as a set, with classes color‐coded after analysis. Visualization of the scores plot reveals any patterns in the data, where the closer two points are, the more similar they are spectrally, and therefore biochemically, and vice versa. Univariate analysis of 1/full width and half height of the phosphate peak was performed to determine the crystallinity of the phosphate peaks.

##### XRD

XRD patterns were recorded using a Rigaku SmartLab powder diffractometer with a 2*θ* scattering range of 5 to 60° and a resolution of 0.1°.

##### FTIR Spectroscopy

All spectra were recorded using an Agilent Technologies Cary 630 FTIR instrument (Agilent Technologies Ltd., Cheadle, UK) at a resolution of 1 cm^−1^ and were an average of 16 scans.

##### SEM

Prior to imaging, the samples were sputter coated with a 10 nm layer of gold. The structures were observed using either a JEOL JSM − 6390 L V operating at 15 kV or a JEOL JSM 7800 F scanning electron microscope (JEOL, Welwyn Garden City, UK) operating at 10–15 kV.

##### EDX Spectroscopy

For qualitative EDX analysis, the samples were sputter coated with a layer of gold (60 s, 20 mA, 8 × 10^−2^ mBar, ≈5 nm) using a Quorum Q150RES sputter coater (Quorum Technologies Ltd) and then investigated using a field‐emission SEM JEOL JSM 7800 F with an EDX system (X‐Max50, Oxford Instruments, Abingdon, UK) at a 10 mm working distance and 10 kV voltage mounted on a brass JEOL holder with 25 mm carbon tabs (G3348N, Agar Scientific, Stansted, UK). Three measurements were performed per sample, and average results are presented.

##### SECM UME Preparation and Characterization

SECM UMEs were prepared using a method described elsewhere.^[^ ^91^ ^]^ Briefly, a borosilicate glass capillary (Sutter Instruments) was pulled at its center using an electric puller (Narishige, model #PP‐100). This resulted in two tapered capillaries which were then flame sealed with a butane torch. Subsequently, a 1.0–1.5 cm length of 7 μm diameter carbon fiber was inserted into the open end of the capillary and pushed into place at the tapered end. The open end was attached to a vacuum line, and the carbon fiber was annealed in place using the electric puller. Next, the UME was polished by using 12, 9, 3, 1, and finally 0.3 μm alumina oxide polishing pads (Buehler). Activated charcoal was then added to the open end of the capillary followed by a Cu wire which was connected to the working electrode lead of the potentiostat.

UMEs were characterized by cyclic voltammetry (CV) and by performing PACs towards an insulating glass substrate in a 0.9 mM ferrocene methanol (FcCH_2_OH) and 10 mM KCl aqueous solution. Figure S12A, Supporting Information, shows the CV obtained with the UME positioned far away (>100 μm tip‐to‐substrate distance) from the substrate. During the forward scan from ≈0.00 to 0.30 V, the recorded sigmoidal signal with a half‐wave potential (*E*_1/2_) at roughly 0.10 V is in good agreement with previous reports for reversible ferrocene oxidation at an inlaid‐disc UME.^[^ ^91^, ^92^ ^]^ The CV demonstrates good reversibility and only a small degree of hysteresis, that is, spacing or lack of overlap in the forward and reverse sigmoidal curve. Figure S12B, Supporting Information, shows the CV converted into the natural logarithmic form using Equation (1)^[^ ^93^ ^]^

(1) $$E=E_{1/2}+\frac{RT}{nF}\ln\left(\frac{i}{i_{\text{ss}}-i}\right)$$

where *i*_ss_ is the steady‐state current achieved at ≈0.25 V in Figure S12A, Supporting Information, *R* is the universal gas constant, *T* is the absolute temperature, *F* is Faraday's constant, and *n* is the number of electrons transferred which was assumed to be 1. The rising portion of the current signal was fit using a linear function providing a slope of 0.032 V. This is close to the expected (*RT*)/*F* = [(8.314 J mol K^−1^)(298.15 K)]/(96485.33 C mol^−1^) = 0.0257 J C^−1^ ≈ 0.026 V. Thus, the UME is reasonably electroactive and conductive.

Figure S12B, Supporting Information, shows the PAC in which the current and tip‐to‐substrate distance have been normalized. In the case of the latter, the normalized distance, *L*, is equal to *d*/*r*_a_, in which *d* is the actual tip‐to‐substrate distance, while *r*_a_ is the radius of the carbon fiber, assumed to be ≈3.5 μm. The normalized PAC was fit using the analytical solution developed by Cornut and Lefrou for PACs over an insulating substrate^[^ ^94^, ^95^ ^]^ and given below in equation 2

(2) iiss≈[2.08Rg0.358(L−0.145Rg)+0.1585]×[2.08Rg0.358(L+0.0023Rg)+1.57+lnRgL+2πRgln(1+πRg2L)]−1

in which *R*_g_ = *r*_g_/*r*_a_, where *r*_g_ is the radius of the insulating glass layer surrounding the electroactive carbon fiber inlaid disc. Equation (2) was applied as a curve‐fitting function in the Igor Pro software version 8; see the dashed, red trace in Figure S12B, Supporting Information, which demonstrated a good fit towards the experimental PAC. An *R*_g_ ≈ 10 was determined using this approach.

The *R*_g_ was also determined using a finite element analysis simulation built in the Comsol Multiphysics software version 6.0. Similar simulations have been described by Zhu et al.^[^ ^96^ ^]^

A simulated PAC is overlaid onto the experimental one shown in Figure S12B, Supporting Information. The *R*_g_ in the simulation was varied iteratively and overlaid onto the experimental curve. The simulation with an *R*_g_ ≈ 10 was determined to have the best match to the experimental one as shown in the red, circle‐marker trace in Figure S12B, Supporting Information.

##### SECM Measurements

SECM measurements were carried out using a custom‐built SECM setup. A CH Instruments potentiostat (Model#6059) was used for controlling the DC potential and recording the current, while a NanoMax 3‐axis positioning system equipped with closed‐loop piezo stepper motors controlled by a BSC‐203 motion controller (Thorlabs) was employed for sample positioning. SECM imaging was performed in feedback mode using ferrocenemethanol (FcMeOH), as a redox mediator, in a two‐electrode configuration with the potential biased between a 7 μm‐diameter carbon fiber UME biased to oxidize FeMeOH versus a Ag wire counter/quasireference electrode. UME fabrication and characterization are fully described above.^[^ ^97^ ^]^ A constant UME tip height of ≈1 μm was maintained throughout as confirmed via PACs performed at 1 μm s^−1^. Tendon samples were mounted onto glass slides using a two‐part epoxy ensuring that the surface of the sample was clear and allowed to cure overnight.

## Supporting Information

Supporting Information is available from the Wiley Online Library or from the author. The Supporting Information contains: Figure S1 (X‐ray diffractograms of samples studied herein), Figure S2 (FTIR spectra of tendons studied herein), Figures S3‐S11 (SEM‐EDX overlays and EDX spectra) and Figure S12 (electrochemical analysis).

## Conflicts of Interest

The authors declare no conflict of interest.

## Author Contributions

**Rebecca F. Shepherd**: data curation (supporting); formal analysis (supporting); investigation (lead); methodology (supporting); writing—original draft (supporting); writing—review and editing (supporting). **Hanaa A. Galeb**: data curation (supporting); funding aquisition (supporting); investigation (supporting); writing—original draft (supporting); writing—review and editing (supporting). **Jade Bentham**: data curation (supporting); investigation (supporting); methodology (supporting); writing—original draft (supporting); writing—review and editing (supporting). **Katelyn Ryan**: investigation (supporting); writing—original draft (supporting); writing—review and editing (supporting). **Nur Adeelah Che Ahmad Tantowi**: investigation (supporting); writing—original draft (supporting); writing—review and editing (supporting). **Sara J. Baldock**: data curation (supporting); investigation (supporting); writing—original draft (supporting); writing—review and editing (supporting). **Nathan R. Halcovitch**: data curation (supporting); investigation (supporting); writing—original draft (supporting); writing—review and editing (supporting). **John G. Hardy**: conceptualization (lead); data curation (lead); formal analysis (lead); funding acquisition (lead); investigation (supporting); methodology (supporting); project administration (lead); supervision (lead); writing—original draft (lead); writing—review and editing (lead). **T. Jane stockmann**: formal analysis (supporting); funding acquisition (supporting); investigation (supporting); methodology (supporting); supervision (supporting); writing—original draft (supporting); writing—review and editing (supporting). **Jemma G. Kerns**: conceptualization (supporting); data curation (supporting); formal analysis (supporting); funding acquisition (supporting); investigation (supporting); methodology (supporting); project administration (supporting); supervision (supporting); writing—original draft (supporting); writing—review and editing (supporting). **Adam M. Taylor**: conceptualization (supporting); data curation (supporting); formal analysis (supporting); funding acquisition (supporting); investigation (supporting); methodology (supporting); project administration (supporting); supervision (supporting); writing—original draft (supporting); writing—review and editing (supporting).

## Supporting information

Supplementary Material

## References

[1] F. Solano , New J. Sci. 2014, 498276, 10.1155/2014/498276.

[2] M. A. Maranduca , D. Branisteanu , D. N. Serban , D. C. Branisteanu , G. Stoleriu , N. Manolache , I. L. Serban , Oncol. Lett. 2019, 17, 4183. 30944614 10.3892/ol.2019.10071 PMC6444329

[3] D. Cao , S. Gong , J. Yang , W. Li , Y. Ge , Y. Wei , Comp. Biochem. Physiol. B 2018, 217, 79. 29269034 10.1016/j.cbpb.2017.12.011

[4] N. E.‐A. El‐Naggar , W. I. Saber , Polymers 2022, 14, 1339. 35406213 10.3390/polym14071339 PMC9002885

[5] J. Park , H. Moon , S. Hong , Biomater. Res. 2019, 23, 24. 31827881 10.1186/s40824-019-0175-9 PMC6889561

[6] M. Kohri , Sci. Technol. Adv. Mater. 2020, 21, 833. 10.1080/14686996.2020.1852057 PMC7832497 33536837

[7] J. D. Simon , D. N. Peles , Acc. Chem. Res. 2010, 43, 1452. 20734991 10.1021/ar100079y

[8] S. Ito , K. Wakamatsu , Pigment Cell Res. 2003, 16, 523. 12950732 10.1034/j.1600-0749.2003.00072.x

[9] S. Ito , K. Wakamatsu , Photochem. Photobiol. 2008, 84, 582. 18435614 10.1111/j.1751-1097.2007.00238.x

[10] V. J. Hearing , J. Invest. Dermatol. 2011, 131, E8. 22094404 10.1038/skinbio.2011.4 PMC6944209

[11] J. A. Swift , Int. J. Cosmet. Sci. 2009, 31, 143. 19175429 10.1111/j.1468-2494.2008.00488.x

[12] S. E. Albers , S. J. Brozena , L. Frank Glass , N. A. Fenske , J. Am. Acad. Dermatol. 1992, 27, 609. 1401313 10.1016/0190-9622(92)70230-d

[13] C. Phornphutkul , W. J. Introne , M. B. Perry , I. Bernardini , M. D. Murphey , D. L. Fitzpatrick , P. D. Anderson , M. Huizing , Y. Anikster , L. H. Gerber , New Eng. J. Med. 2002, 347, 2111. 12501223 10.1056/NEJMoa021736

[14] A. Zatkova , L. Ranganath , L. Kadasi , Appl. Clin. Genet. 2020, 13, 37. 32158253 10.2147/TACG.S186773 PMC6986890

[15] L. R. Ranganath , J. C. Jarvis , J. A. Gallagher , J. Clin. Pathol. 2013, 66, 367. 23486607 10.1136/jclinpath-2012-200877

[16] J. B. Mistry , M. Bukhari , A. M. Taylor , Rare Diseases 2013, 1, e27475. 25003018 10.4161/rdis.27475 PMC3978898

[17] M. S. Milella , M. Geminiani , A. Trezza , A. Visibelli , D. Braconi , A. Santucci , Cells 2024, 13, 1072. 38920699 10.3390/cells13121072 PMC11201470

[18] G. Bernardini , D. Braconi , A. Zatkova , N. Sireau , M. J. Kujawa , W. J. Introne , O. Spiga , M. Geminiani , J. A. Gallagher , L. R. Ranganath , A. Santucci , Nat. Rev. Dis. Primers 2024, 10, 16. 38453957 10.1038/s41572-024-00498-x

[19] F. A. Zucca , A. Capucciati , C. Bellei , M. Sarna , T. Sarna , E. Monzani , L. Casella , L. Zecca , IUBMB Life 2023, 75, 55. 35689524 10.1002/iub.2654 PMC10084223

[20] K. Tajima , D. Yamanaka , K. I. Ishibashi , Y. Adachi , N. Ohno , FEBS Open Bio 2019, 9, 791. 10.1002/2211-5463.12615 PMC6443868 30984552

[21] F. Solano , Int. J. Mol. Sci. 2017, 18, 1561.28718807

[22] S. Park , C. Lee , J. Lee , S. Jung , K.‐Y. Choi , Biotechnol. Bioproc. Eng. 2020, 25, 646.

[23] F. Scognamiglio , A. Travan , G. Turco , M. Borgogna , E. Marsich , M. Pasqua , S. Paoletti , I. Donati , Coll. Surf. B 2017, 155, 553. 10.1016/j.colsurfb.2017.04.057 28499217

[24] A. M. Taylor , M.‐F. Hsueh , L. R. Ranganath , J. A. Gallagher , J. P. Dillon , J. L. Huebner , J. B. Catterall , V. B. Kraus , Rheumatology 2017, 56, 156. 28028161 10.1093/rheumatology/kew355 PMC5188995

[25] A. M. Taylor , B. Wlodarski , I. A. Prior , P. J. Wilson , J. C. Jarvis , L. R. Ranganath , J. A. Gallagher , Rheumatology 2010, 49, 1412. 20181670 10.1093/rheumatology/keq027

[26] L. Wu , H. Shao , Z. Fang , Y. Zhao , C. Y. Cao , Q. Li , ACS Biomater. Sci. Eng. 2019, 5, 4272. 33417783 10.1021/acsbiomaterials.9b00593

[27] K. Ikoma , M. Kido , M. Nagae , T. Ikeda , T. Shirai , K. Ueshima , Y. Arai , R. Oda , H. Fujiwara , T. Kubo , J. Orthop. Res. 2013, 31, 1708. 23832876 10.1002/jor.22424

[28] E. Yamamoto , K. Hayashi , N. Yamamoto , Clin. Biomech. 1999, 14, 418. 10.1016/s0268-0033(99)00006-6 10521624

[29] A. P. Rumian , E. R. C. Draper , A. L. Wallace , A. E. Goodship , Bone Jt. J. 2009, 91‐B, 557. 10.1302/0301-620X.91B4.21580 19336822

[30] L. Ranganath , M. Khedr , A. Mistry , S. Vinjamuri , J. Gallagher , Bone Rep. 2021, 15, 101151. 34926730 10.1016/j.bonr.2021.101151 PMC8649650

[31] A. Taylor , A. Boyde , P. J. Wilson , J. C. Jarvis , J. S. Davidson , J. Hunt , L. Ranganath , J. A. Gallagher , Arthritis Rheum. 2011, 63, 3887. 22127706 10.1002/art.30606

[32] M. L. Chen , J. W. Ruberti , T. D. Nguyen , J. Mech. Behav. Biomed. Mater. 2018, 82, 345. 29655120 10.1016/j.jmbbm.2018.03.027

[33] R. G. Wells , Elife 2022, 11, e77041.35188461

[34] W. Cao , X. Zhou , N. C. McCallum , Z. Hu , Q. Z. Ni , U. Kapoor , C. M. Heil , K. S. Cay , T. Zand , A. J. Mantanona , A. Jayaraman , A. Dhinojwala , D. D. Deheyn , M. D. Shawkey , M. D. Burkart , J. D. Rinehart , N. C. Gianneschi , J. Am. Chem. Soc. 2021, 143, 2622. 33560127 10.1021/jacs.0c12322

[35] A. A. Putnam , J. J. Wilker , Soft Matter 2021, 17, 1999. 33438707 10.1039/d0sm01944e

[36] M. Dunbar , S. Keten , Macromolecules 2020, 53, 9397.

[37] X. Hu , Z. Li , Z. Yang , F. Zhu , W. Zhao , G. Duan , Y. Li , ACS Macro Lett. 2022, 11, 251. 35574777 10.1021/acsmacrolett.1c00729

[38] H. A. Galeb , A. Lamantia , A. Robson , K. König , J. Eichhorn , S. J. Baldock , M. D. Ashton , J. V. Baum , R. L. Mort , B. J. Robinson , F. H. Schacher , V. Chechik , A. M. Taylor , J. G. Hardy , Macromol. Chem. Phys. 2022, 223, 2100489.

[39] H. A. Galeb , J. Eichhorn , S. Harley , A. J. Robson , L. Martocq , S. J. Nicholson , M. D. Ashton , H. A. M. Abdelmohsen , E. Pelit , S. J. Baldock , N. R. Halcovitch , B. J. Robinson , F. H. Schacher , V. Chechik , K. Vercruysse , A. M. Taylor , J. G. Hardy , Macromol. Chem. Phys. 2023, 224, 2300025.

[40] M. R. Buckley , E. B. Evans , P. E. Matuszewski , Y.‐L. Chen , L. N. Satchel , D. M. Elliott , L. J. Soslowsky , G. R. Dodge , Connect Tissue Res. 2013, 54, 374. 24088220 10.3109/03008207.2013.847096 PMC6056177

[41] I. Tresoldi , F. Oliva , M. Benvenuto , M. Fantini , L. Masuelli , R. Bei , A. Modesti , Muscles Ligaments Tendons J. 2013, 3, 2. 23885339 10.11138/mltj/2013.3.1.002 PMC3676160

[42] R. W. Taylor , G. K. Mitchell , J. L. Andrade , K. K. Svoboda , Anat. Rec. 2020, 303, 1664. 10.1002/ar.24091 30768858

[43] W. Traub , T. Arad , S. Weiner , Proc. Natl. Acad. Sci. 1989, 86, 9822. 2602376 10.1073/pnas.86.24.9822 PMC298594

[44] N. Almora‐Barrios , N. H. de Leeuw , CrystEngComm 2010, 12, 960.

[45] N. Almora‐Barrios , N. H. de Leeuw , Langmuir 2010, 26, 14535. 20731400 10.1021/la101151e

[46] M. Cutini , M. Corno , D. Costa , P. Ugliengo , J. Phys. Chem. C. 2019, 123, 7540.

[47] E. L. Mertz , S. Leikin , Biochemistry 2004, 43, 14901. 15554697 10.1021/bi048788b

[48] A. Büngeler , B. Hämisch , O. I. Strube , Int. J. Mol. Sci. 2017, 18, 1901. 28878140 10.3390/ijms18091901 PMC5618550

[49] H. A. Galeb , E. L. Wilkinson , A. F. Stowell , H. Lin , S. T. Murphy , P. L. Martin‐Hirsch , R. L. Mort , A. M. Taylor , J. G. Hardy , Global Challenges 2021, 5, 2000102. 33552556 10.1002/gch2.202000102 PMC7857133

[50] W. Y. Chow , A. M. Taylor , D. G. Reid , J. A. Gallagher , M. J. Duer , J. Inherited Metab. Dis. 2011, 34, 1137. 21735270 10.1007/s10545-011-9373-x

[51] A. M. Taylor , D. D. Jenks , V. D. Kammath , B. P. Norman , J. P. Dillon , J. A. Gallagher , L. R. Ranganath , J. G. Kerns , Osteoarthritis Cartilage 2019, 27, 1244. 31022456 10.1016/j.joca.2019.04.012

[52] M. Nicolai , G. Gonçalves , F. Natalio , M. Humanes , J. Inorg. Biochem. 2011, 105, 887. 21507323 10.1016/j.jinorgbio.2011.03.014

[53] S. N. Dezidério , C. A. Brunello , M. I. N. da Silva , M. A. Cotta , C. F. O. Graeff , J. Non‐Cryst. Solids 2004, 338, 634.

[54] V. Capozzi , G. Perna , P. Carmone , A. Gallone , M. Lastella , E. Mezzenga , G. Quartucci , M. Ambrico , V. Augelli , P. J. Biagi , T. Ligonzo , A. Minafra , L. Schiavulli , M. Pallara , R. Cicero , Thin Solid Films 2006, 511, 362.

[55] G. Khouqeer , M. Alghrably , N. Madkhali , M. Dhahri , M. Jaremko , A.‐H. Emwas , Nano Select 2022, 3, 1598.

[56] A. K. Maurya , A. Parrilli , T. Kochetkova , J. Schwiedrzik , A. Dommann , A. Neels , Acta Biomater. 2021, 129, 169. 34052502 10.1016/j.actbio.2021.05.022

[57] A. Spilotros , A. Gruzinov , D. I. Svergun , Encyclopedia of Analytical Chemistry, John Wiley & Sons, Ltd. 2019, pp. 1–39, 10.1002/9780470027318.a9674.

[58] J. De Meutter , E. Goormaghtigh , Eur. Biophy. J. 2021, 50, 641. 10.1007/s00249-021-01507-7 PMC8189991 33558954

[59] S. J. Gadaleta , W. J. Landis , A. L. Boskey , R. Mendelsohn , Connect. Tissue Res. 1996, 34, 203. 9023049 10.3109/03008209609000699

[60] M. Walters , Y. Leung , N. Blumenthal , K. Konsker , R. LeGeros , J. Inorg. Biochem. 1990, 39, 193. 2168470 10.1016/0162-0134(90)84002-7

[61] S. Gadaleta , N. Camacho , R. Mendelsohn , A. Boskey , Calcif. Tissue Int. 1996, 58, 17. 8825234 10.1007/BF02509541

[62] I. Movre Šapić , A. Vidak , V. Dananić , Bull. Chem. Technol. Bosnia Herzeg 2019, 52, 17.

[63] K. G. Grønlien , M. E. Pedersen , K. W. Sanden , V. Høst , J. Karlsen , H. H. Tønnesen , Sustainable Chem. Pharm. 2019, 13, 100166.

[64] M. B. Fauzi , Y. Lokanathan , B. S. Aminuddin , B. H. I. Ruszymah , S. R. Chowdhury , Mater. Sci. Eng. C 2016, 68, 163. 10.1016/j.msec.2016.05.109 27524008

[65] I. Rehman , W. Bonfield , J. Mater. Sci.: Mater. Med. 1997, 8, 10.1023/A:1018570213546. 15348834

[66] M. Xu , J. Liu , J. Sun , X. Xu , Y. Hu , B. Liu , Orthop. Surg. 2020, 12, 366. 32096911 10.1111/os.12637 PMC7189050

[67] S. Nicholls , L. Gathercole , J. Shah , Ann. Rheum. Dis. 1984, 43, 477. 6742908 10.1136/ard.43.3.477 PMC1001373

[68] P. Sun , F. O. Laforge , M. V. Mirkin , Phys. Chem. Chem. Phys. 2007, 9, 802. 17287874 10.1039/b612259k

[69] L. Huang , Z. Li , Y. Lou , F. Cao , D. Zhang , X. Li , Materials 2018, 11, 1389. 30096895 10.3390/ma11081389 PMC6119995

[70] F. Conzuelo , A. Schulte , W. Schuhmann , Proc. R. Soc. A 2018, 474, 20180409. 30839832 10.1098/rspa.2018.0409 PMC6237495

[71] I. Beaulieu , S. Kuss , J. Mauzeroll , M. Geissler , Anal. Chem. 2011, 83, 1485. 21214262 10.1021/ac101906a

[72] T.‐E. Lin , F. Cortés‐Salazar , A. Lesch , L. Qiao , A. Bondarenko , H. H. Girault , Electrochim. Acta 2015, 179, 57.

[73] N. Gogurla , B. Roy , K. Min , J.‐Y. Park , S. Kim , Adv. Mater. Technol. 2020, 5, 1900936.

[74] M. Caldas , A. C. Santos , F. Veiga , R. Rebelo , R. L. Reis , V. M. Correlo , Acta Biomater. 2020, 105, 26. 32014585 10.1016/j.actbio.2020.01.044

[75] P. Meredith , T. Sarna , Pigment Cell Res. 2006, 19, 572. 17083485 10.1111/j.1600-0749.2006.00345.x

[76] O. Alrawashdeh , M. Alsbou , H. Alzoubi , H. Al‐shagahin , Neurol. Int. 2017, 8. 10.4081/ni.2016.6841 PMC5226046 28217270

[77] S. K. Nanda , D. R. Suresh , A. Vamseedhar , K. Pratibha , B. Arjun , Indian J. Clin. Biochem. 2010, 25, 213. 23105912 10.1007/s12291-010-0038-6 PMC3453108

[78] İ. E. Ertek , S. Candansayar , Psychiatr. Ann. 2020, 50, 81.

[79] M. Galdston , J. M. Steele , K. Dobriner , Am. J. Med. 1952, 13, 432. 12985601 10.1016/0002-9343(52)90298-2

[80] W. Liu , R. A. Prayson , Arch. Path. Lab. 2001, 125, 961. 10.5858/2001-125-0961-DMIIOA 11419988

[81] T. R. Helliwell , J. A. Gallagher , L. Ranganath , Histopathology 2008, 53, 503. 18336562 10.1111/j.1365-2559.2008.03000.x

[82] E. Rezvani Ghomi , N. Nourbakhsh , M. Akbari Kenari , M. Zare , S. Ramakrishna , J. Biomed. Mater. Res. B. 2021, 109, 1986. 10.1002/jbm.b.34881 34028179

[83] A. Veiga , F. Castro , F. Rocha , A. L. Oliveira , J. Biomed. Mater. Res. B 2022, 110, 1192. 10.1002/jbm.b.34976 34860461

[84] B. Lowe , J. G. Hardy , L. J. Walsh , ACS Omega 2020, 5, 10.1021/acsomega.9b02917. PMC6963893 31956745

[85] A. Mavridi‐Printezi , A. Menichetti , D. Mordini , M. Montalti , Int. J. Mol. Sci. 2023, 24, 9689. 37298641 10.3390/ijms24119689 PMC10253489

[86] J. G. Hardy , J. Y. Lee , C. E. Schmidt , Curr. Opin. Biotechnol. 2013, 24, 847. 23578463 10.1016/j.copbio.2013.03.011

[87] J. G. Hardy , R. C. Sukhavasi , D. Aguilar , M. K. Villancio‐Wolter , D. J. Mouser , S. A. Geissler , L. Nguy , J. K. Chow , D. L. Kaplan , C. E. Schmidt , J. Mater. Chem. B. 2015, 3, 8059. 32262862 10.1039/c5tb00714c

[88] Z. Liu , X. Wan , Z. L. Wang , L. Li , Adv. Mater. 2021, 33, 2007429. 10.1002/adma.202007429 34117803

[89] J. Trevisan , P. P. Angelov , P. L. Carmichael , A. D. Scott , F. L. Martin , Analyst 2012, 137, 3202. 22627698 10.1039/c2an16300d

[90] F. L. Martin , J. G. Kelly , V. Llabjani , P. L. Martin‐Hirsch , I. I. Patel , J. Trevisan , N. J. Fullwood , M. J. Walsh , Nat. Prot. 2010, 5, 1748. 10.1038/nprot.2010.133 21030951

[91] C. Santana Santos , B. N. Jaato , I. Sanjuán , W. Schuhmann , C. Andronescu , Chem. Rev. 2023, 123, 4972. 36972701 10.1021/acs.chemrev.2c00766 PMC10168669

[92] D. Polcari , P. Dauphin‐Ducharme , J. Mauzeroll , Chem. Rev. 2016, 116, 13234. 27736057 10.1021/acs.chemrev.6b00067

[93] J. T. Cox , J. P. Guerrette , B. Zhang , Anal. Chem. 2012, 84, 8797. 22992030 10.1021/ac302219p PMC3474901

[94] C. Lefrou , R. Cornut , ChemPhysChem 2010, 11, 547. 20058287 10.1002/cphc.200900600

[95] R. Cornut , C. Lefrou , J. Electroanal. Chem. 2007, 604, 91.

[96] R. Zhu , S. M. Macfie , Z. Ding , Langmuir 2008, 24, 14261. 19360968 10.1021/la8018875

[97] N. Ahmadinasab , T. J. Stockmann , ChemElectroChem 2022, 9, e202200162.

