# Supplementary material for: Analysis of Interactions Between Pyomelanin and the Extracellular Matrix in an Ex Vivo Turkey Tendon Model
Source: ChemistryOpen. 2025 Apr 13;14(6):e202500194. doi: 10.1002/open.202500194 (PMC12138056; doi:10.1002/open.202500194)
Supplement: Supplementary file 1 — Supplementary Material [file OPEN-14-e202500194-s001.pdf]

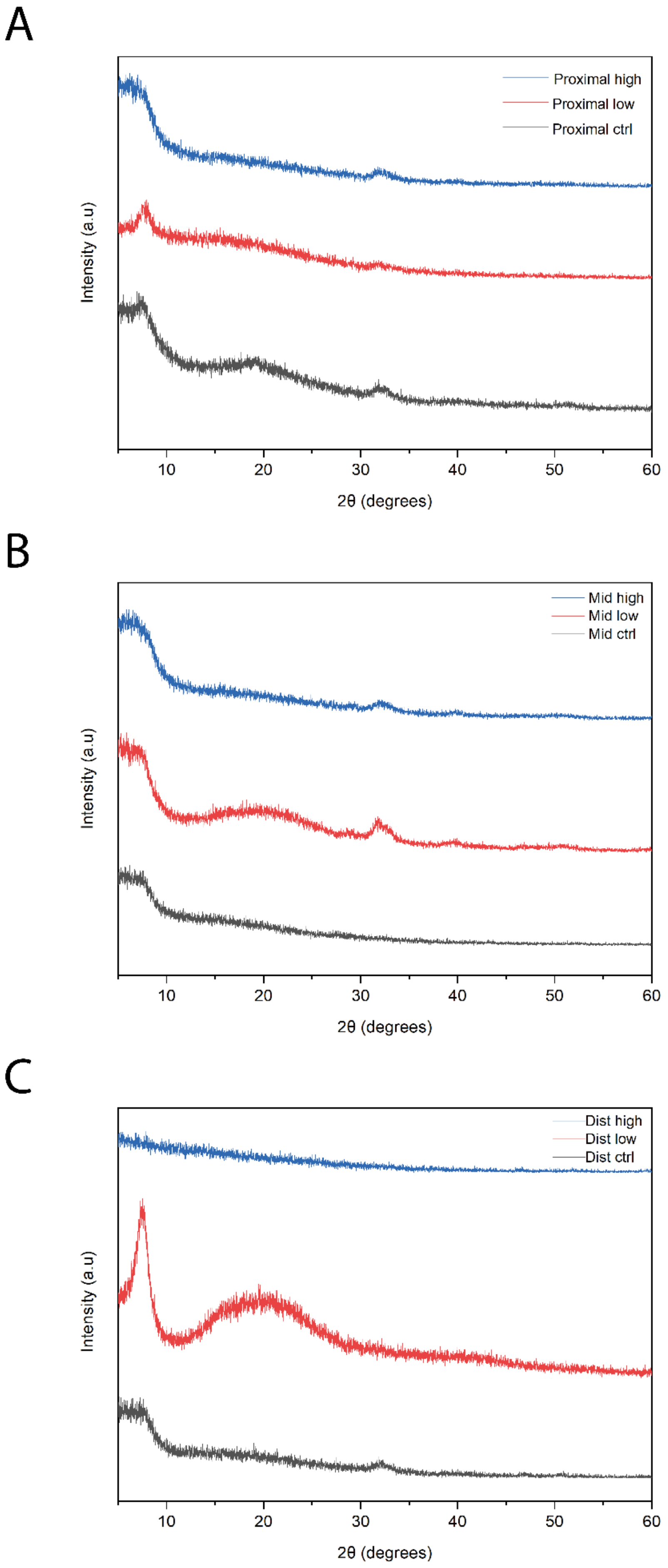

Figure S1. X-ray diffractograms of samples studied herein. A) Proximal section of tendon. B) Mid-point of tendon. C) Distal section of tendon.

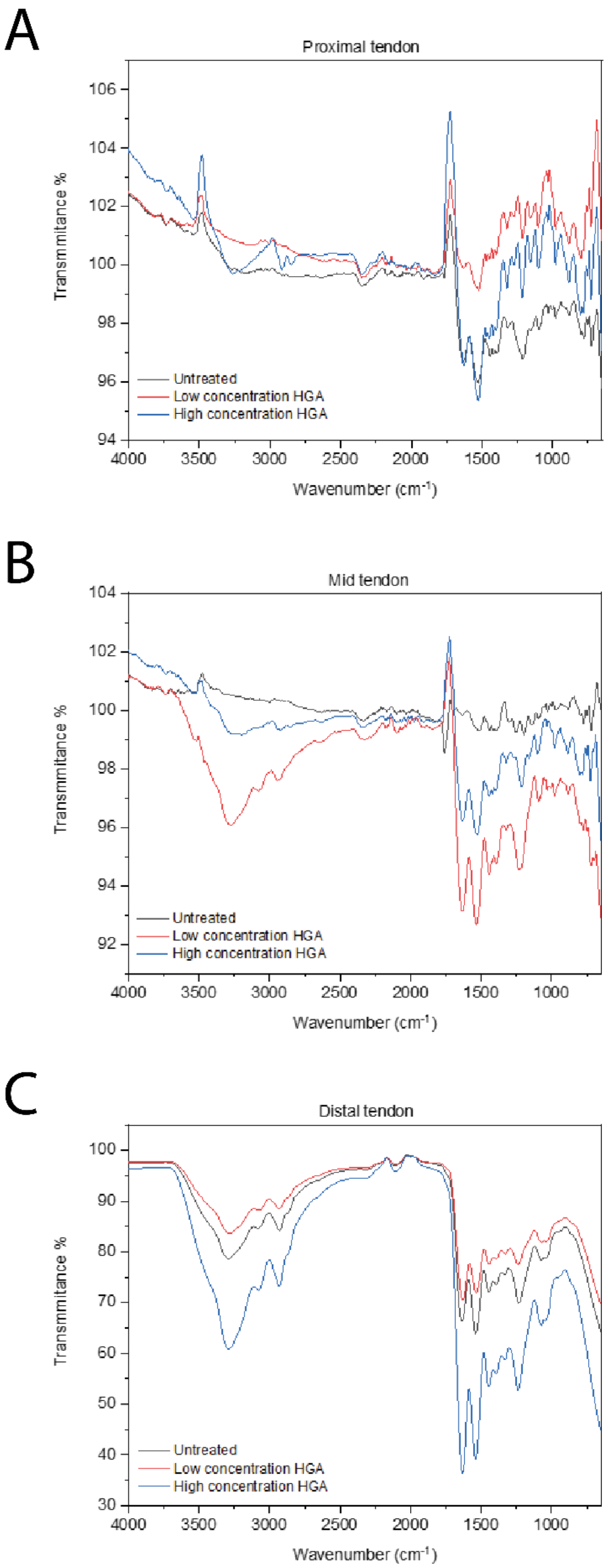

Figure S2. FTIR spectra of tendons studied herein. A) Proximal section of tendon. B) Mid-point of tendon. C) Distal section of tendon.

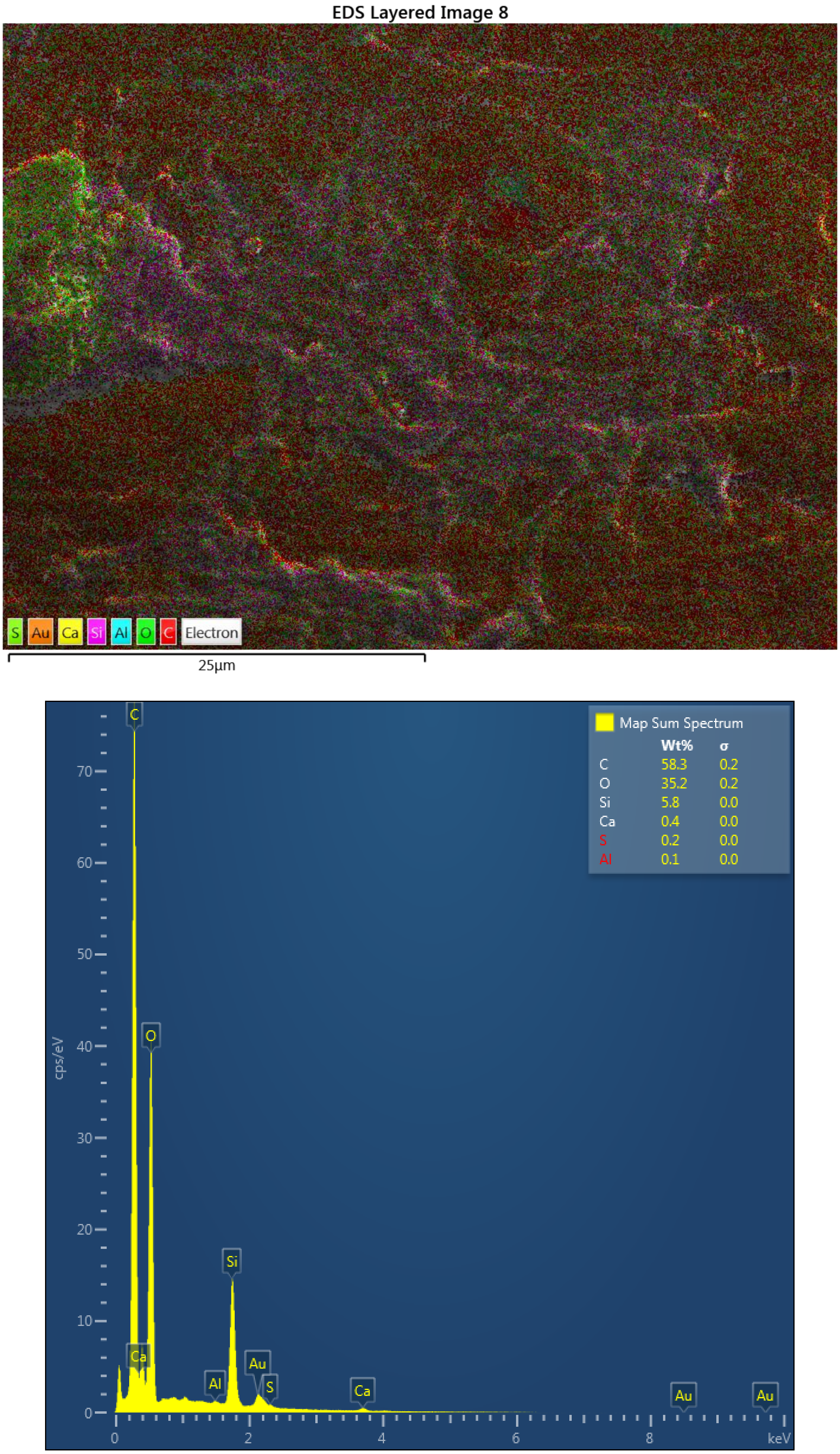

Figure S3. Proximal section of tendon (control). Top) SEM-EDX overlay. Bottom) EDX spectrum.

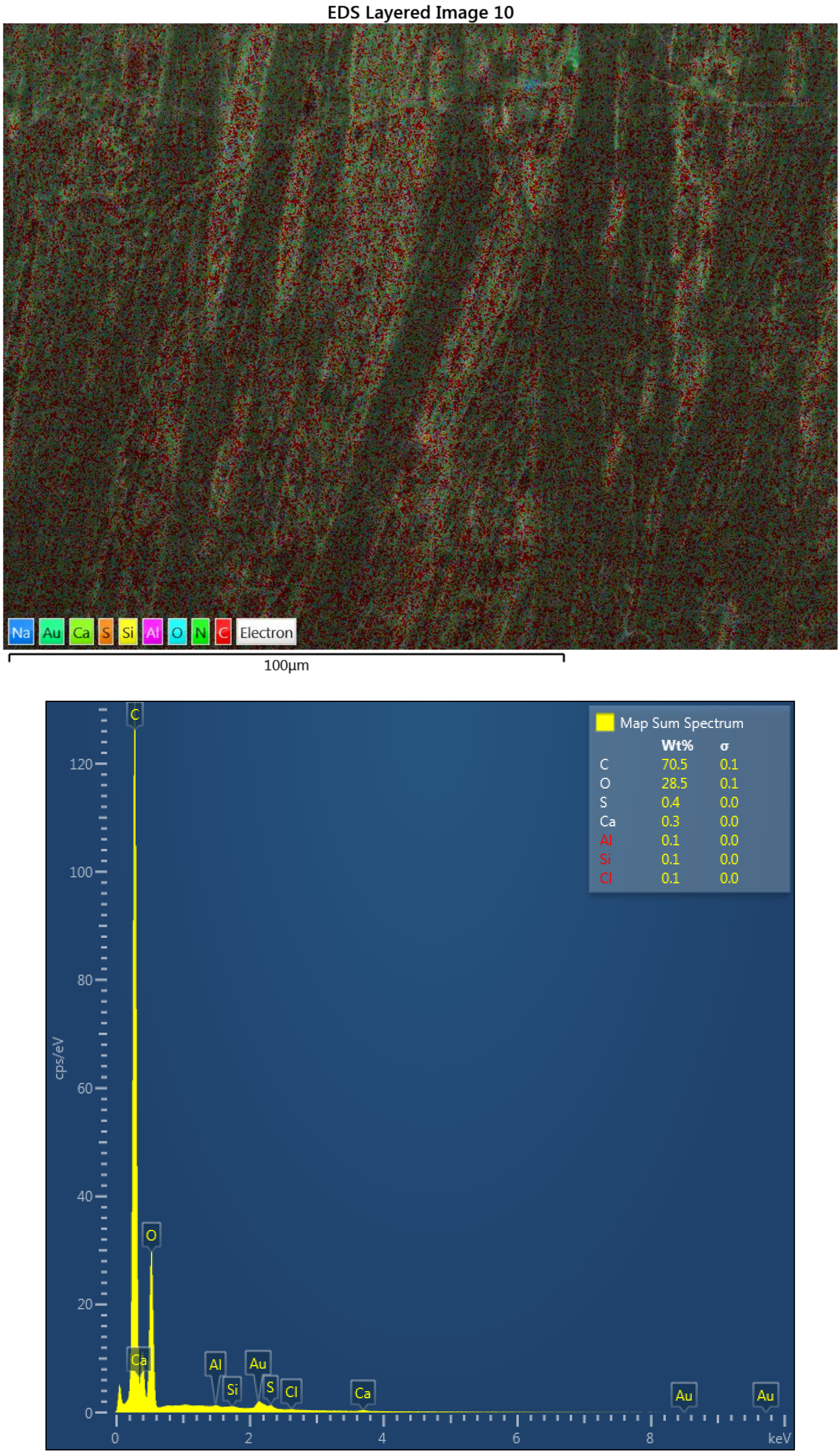

Figure S4. Proximal section of tendon (low [HGA]). Top) SEM-EDX overlay. Bottom) EDX spectrum.

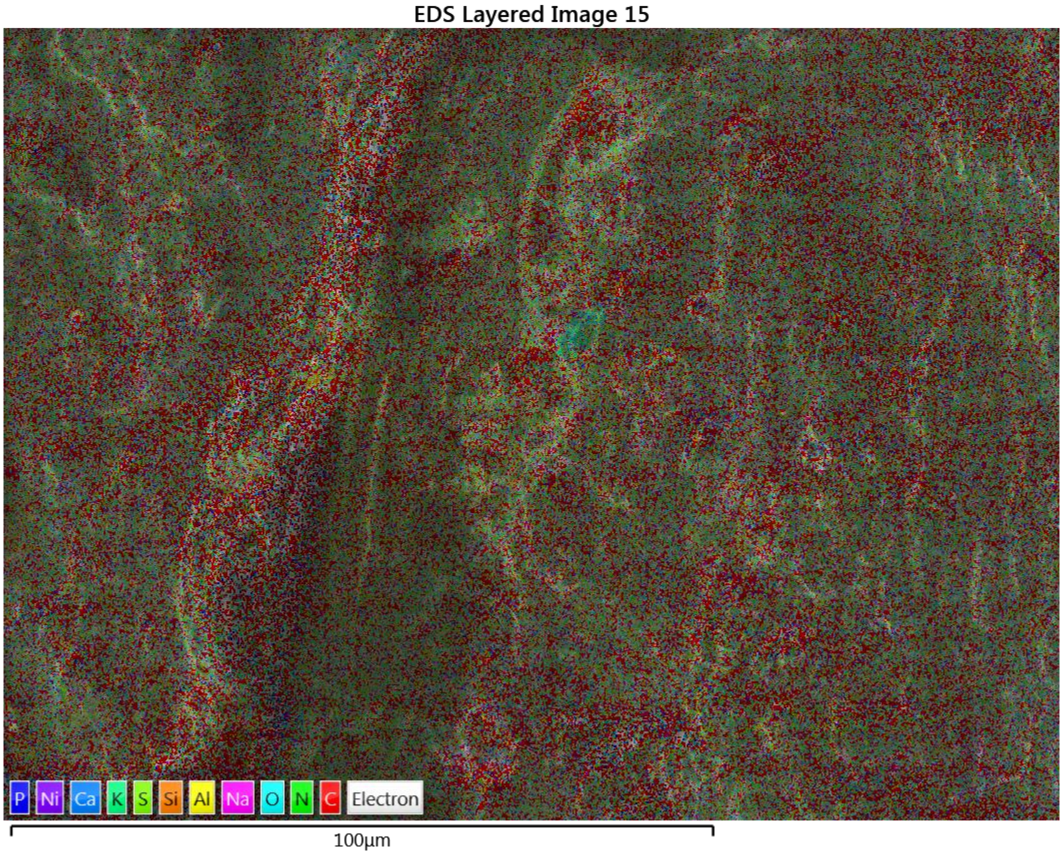

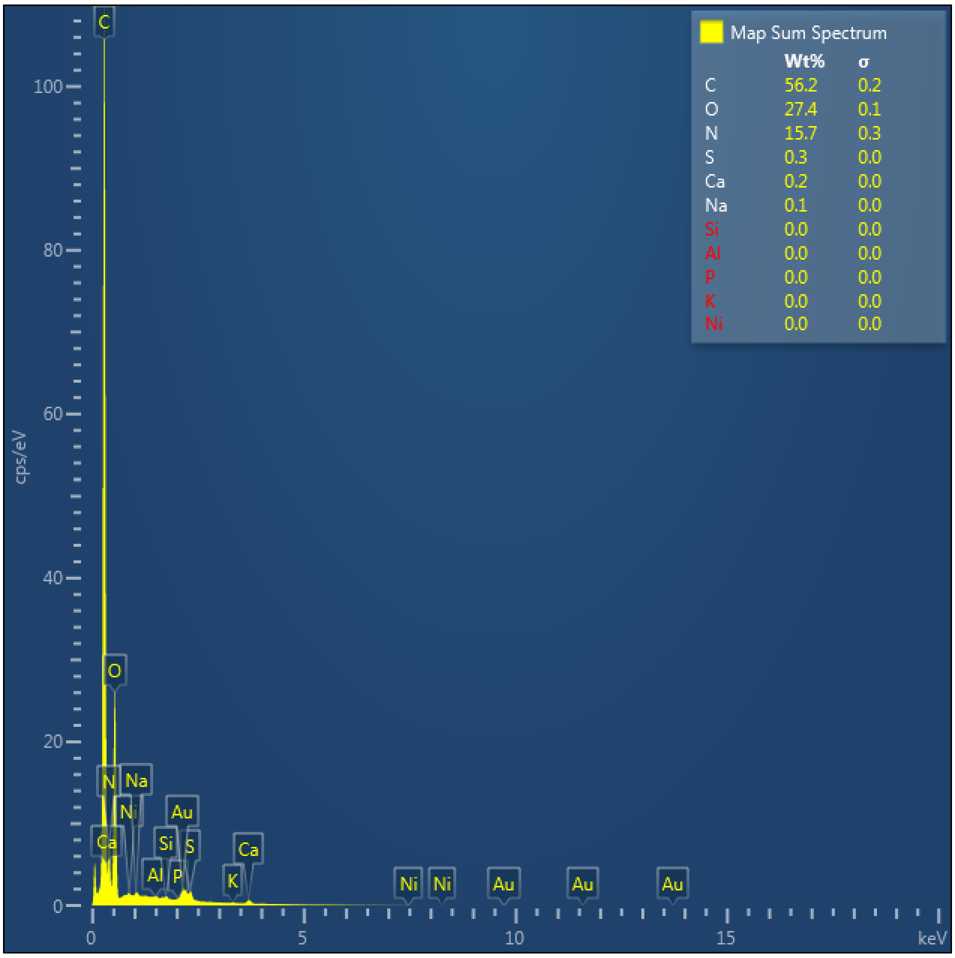

Figure S5. Proximal section of tendon (high [HGA]). Top) SEM-EDX overlay. Bottom) EDX spectrum.

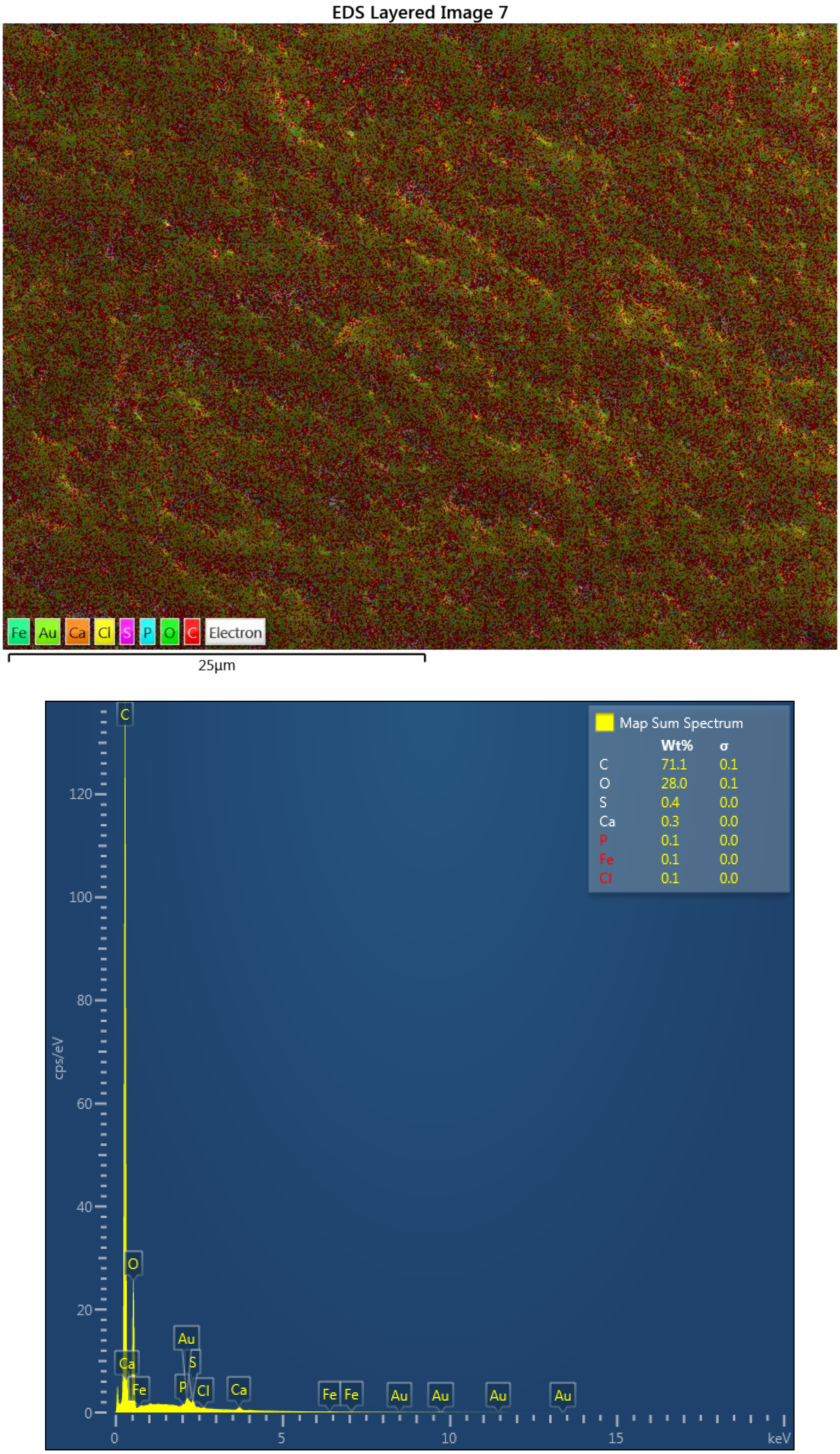

Figure S6. Mid-point section of tendon (control). Top) SEM-EDX overlay. Bottom) EDX spectrum.

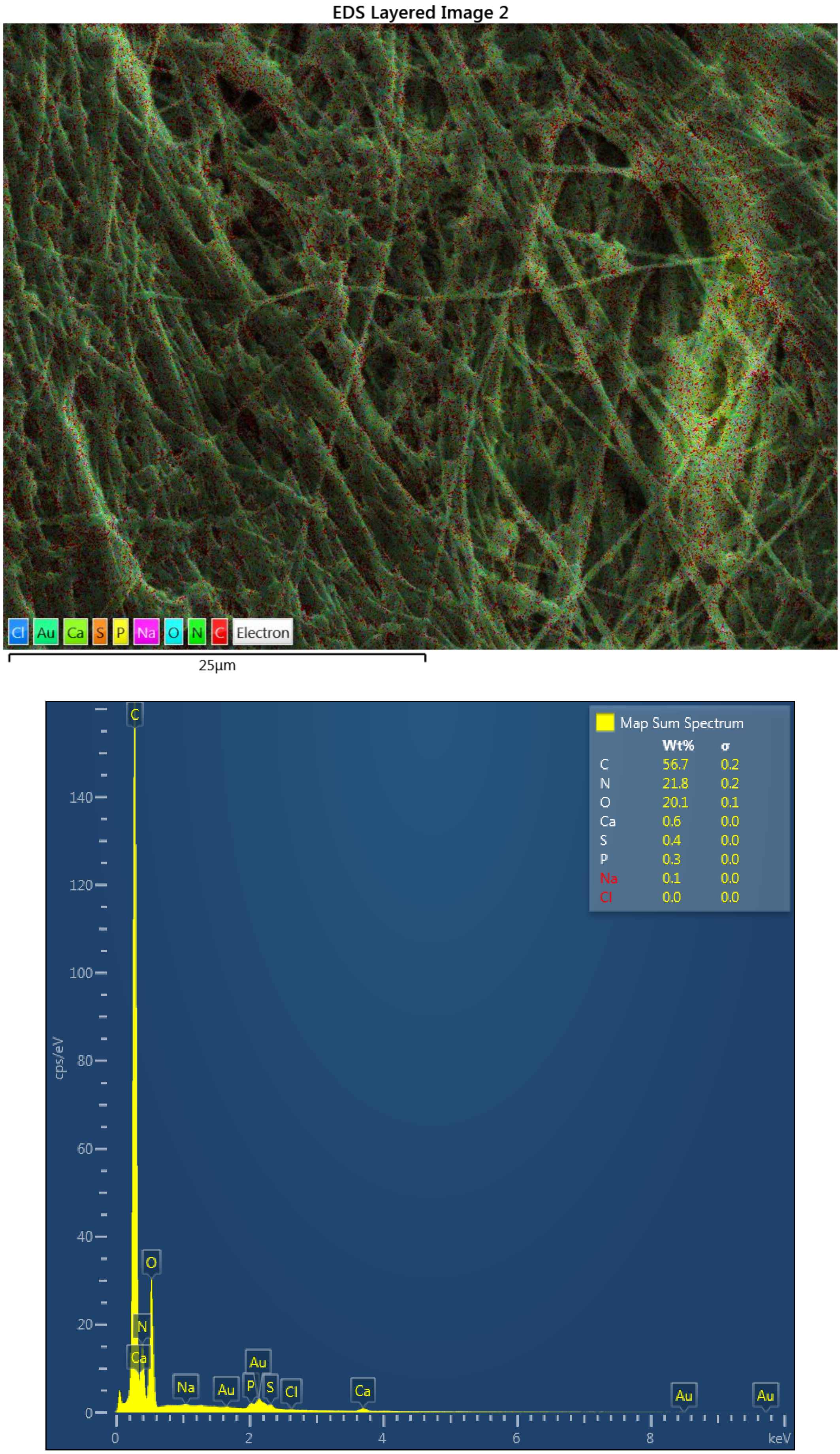

Figure S7. Mid-point section of tendon (low [HGA]). Top) SEM-EDX overlay. Bottom) EDX spectrum.

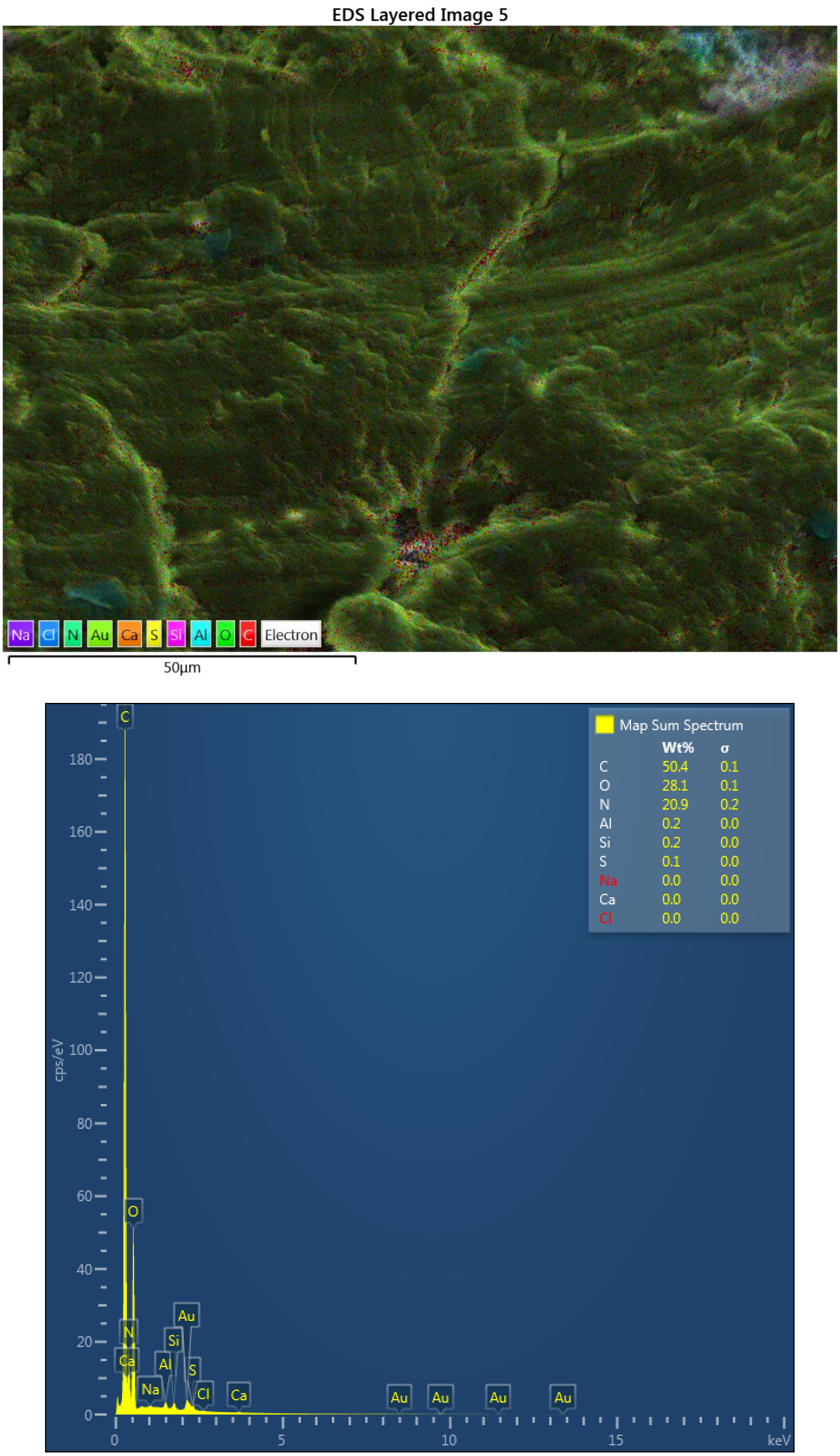

Figure S8. Mid-point section of tendon (high [HGA]). Top) SEM-EDX overlay. Bottom) EDX spectrum.

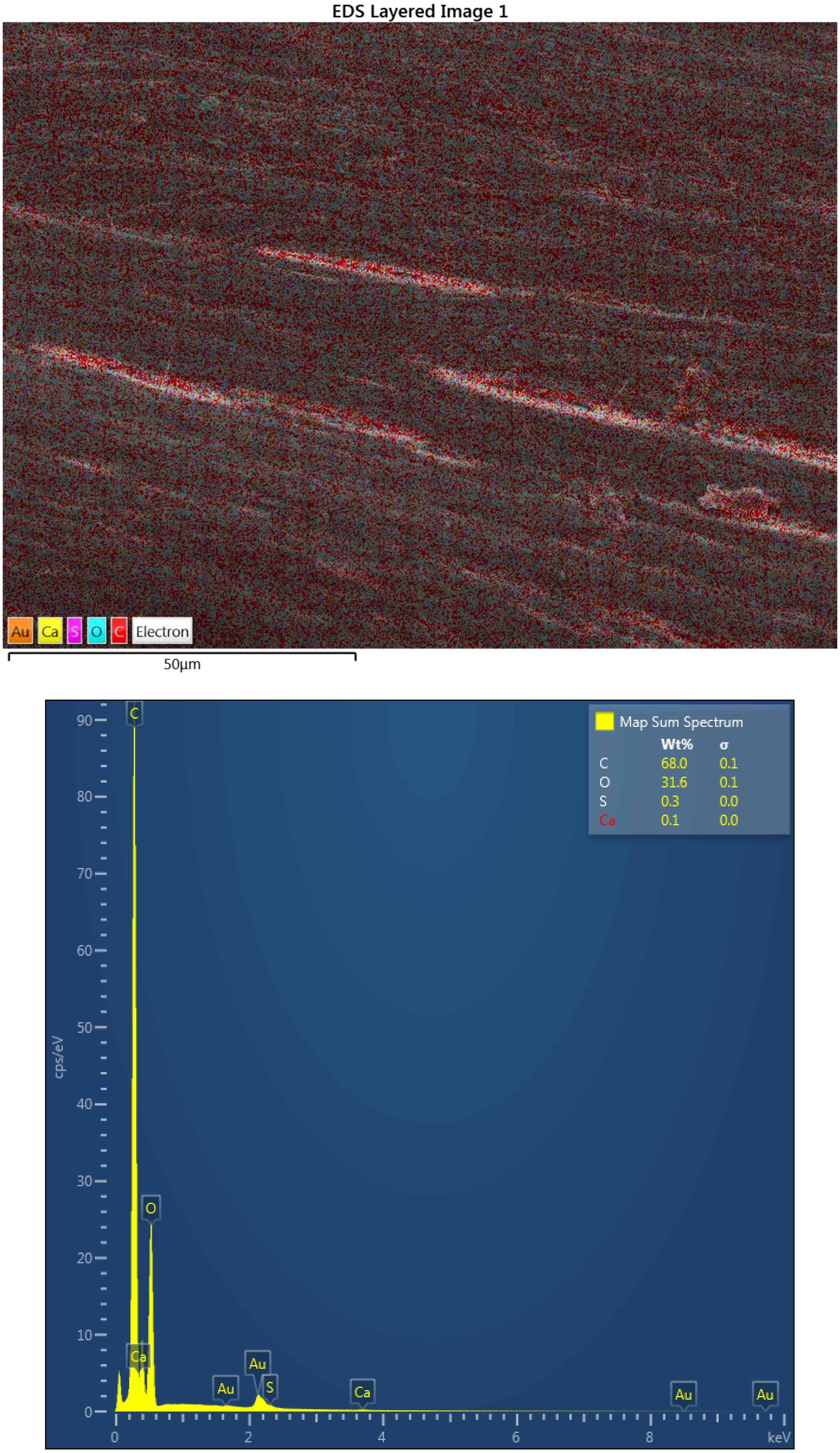

Figure S9. Distal section of tendon (control). Top) SEM-EDX overlay. Bottom) EDX spectrum.

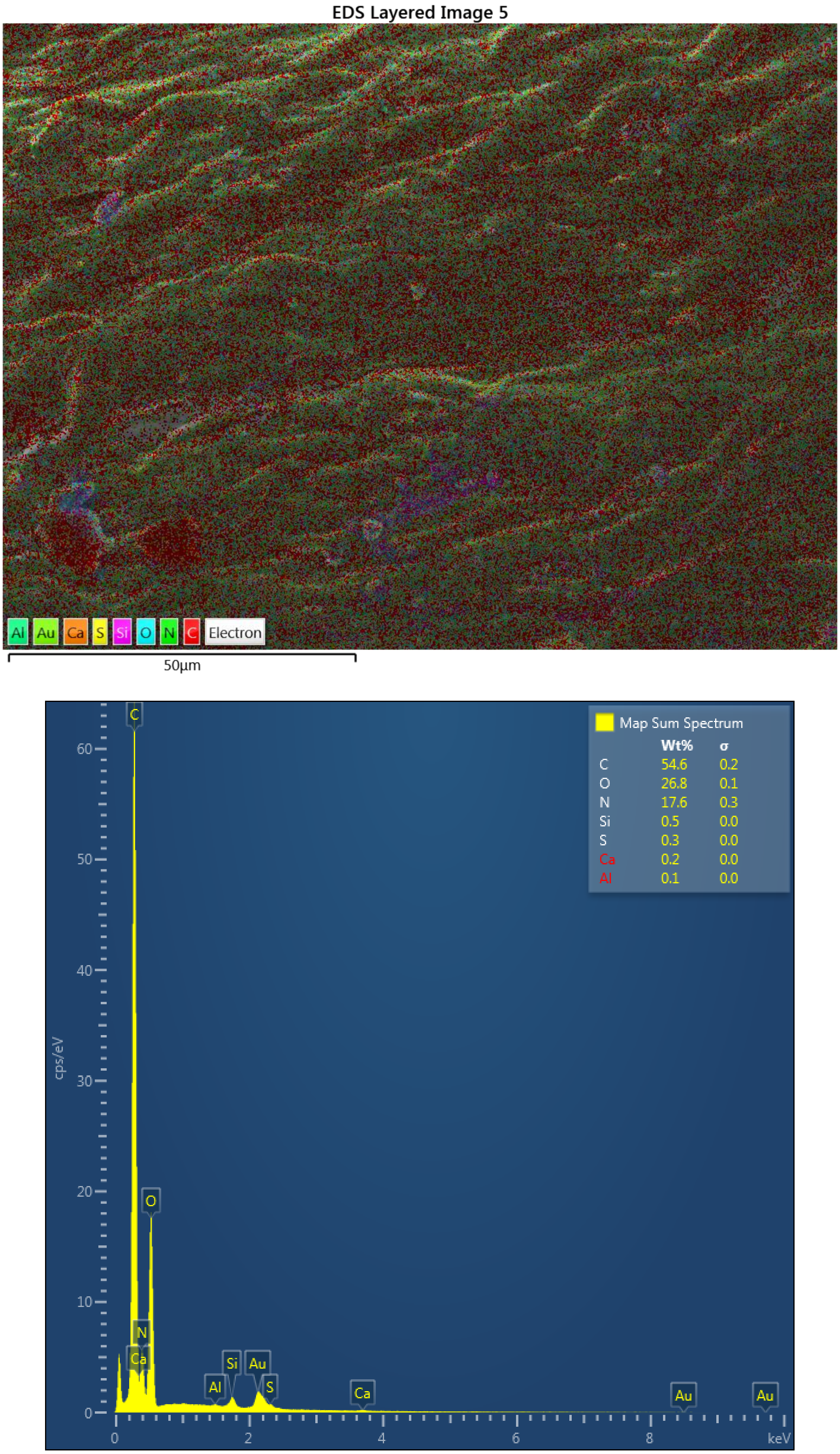

Figure S10. Distal section of tendon (low [HGA]). Top) SEM-EDX overlay. Bottom) EDX spectrum.

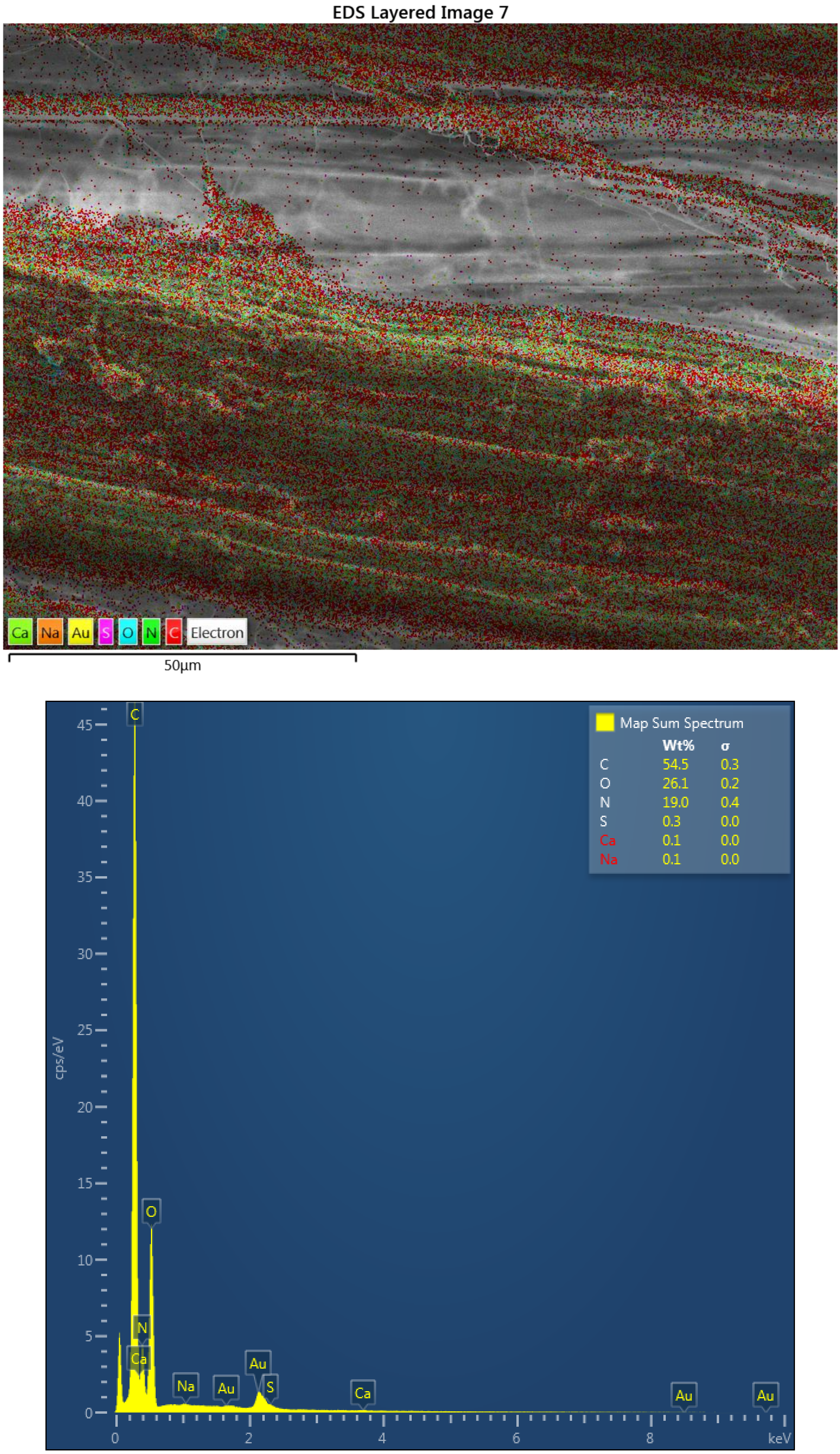

Figure S11. Distal section of tendon (high [HGA]). Top) SEM-EDX overlay. Bottom) EDX spectrum.

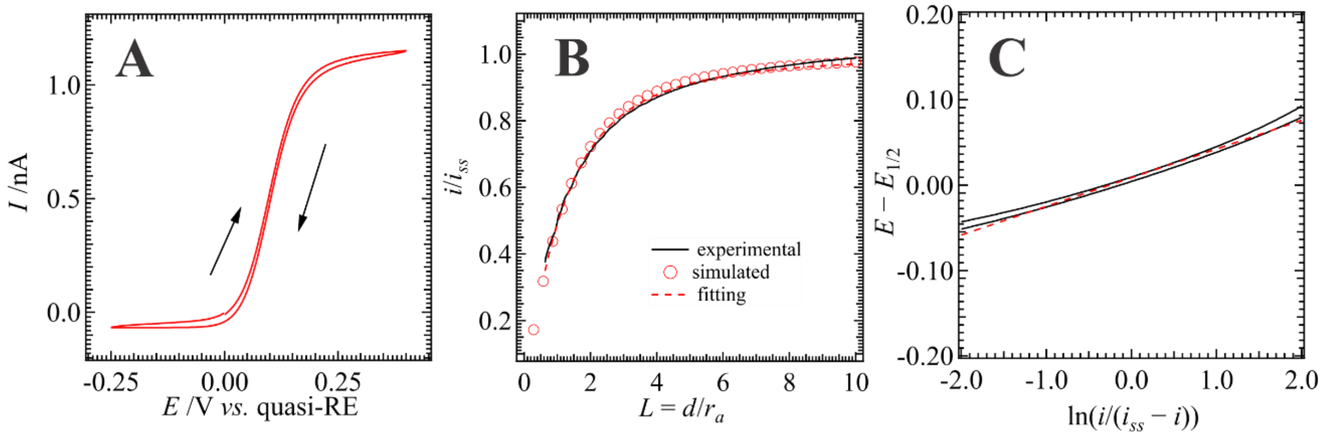

Figure S12. A) Cyclic voltammogram (CV) obtained at 0.02 Vs^{-1} in an aqueous solution of 0.9 mM ferrocene methanol ($FcCH_2OH$) and 10 mM KCl. **B**) Probe approach curve (PAC) performed in the solution described in A towards a glass, insulating substrate (black curve) along with an overlaid PAC simulated in Comsol Multiphysics software version 6.0 and a curve fitting using the analytical solution developed by Cornut and Lefrou. Both the simulation and curve fitting indicate an $R_g = r_g/r_a \approx 10$. PACs were performed at a tip velocity of 1 µm s^{-1}. **C**) The CV in A has been converted into a plot of $(E - E_{1/2})$ versus $\ln(i/(i_{ss} - i))$, black trace, while the red, dashed curve is the linear best fit according to equation 2.
